# Altertoxin II, a Highly Effective and Specific Compound against Ewing Sarcoma

**DOI:** 10.3390/cancers13246176

**Published:** 2021-12-07

**Authors:** Andrew J. Robles, Wentao Dai, Saikat Haldar, Hongyan Ma, Victoria M. Anderson, Ross D. Overacker, April L. Risinger, Sandra Loesgen, Peter J. Houghton, Robert H. Cichewicz, Susan L. Mooberry

**Affiliations:** 1 Department of Pharmacology, The University of Texas Health Science Center at San Antonio, San Antonio, TX 78229, USA; roblesa3@uthscsa.edu (A.J.R.); risingera@uthscsa.edu (A.L.R.); 2 Mays Cancer Center, The University of Texas Health Science Center at San Antonio, San Antonio, TX 78229, USA; houghtonp@uthscsa.edu; 3 Greehey Children’s Cancer Research Institute, The University of Texas Health Science Center at San Antonio, San Antonio, TX 78229, USA; 4 Natural Products Discovery Group, Institute for Natural Products Applications and Research Technologies, and Department of Chemistry & Biochemistry, Stephenson Life Science Research Center, University of Oklahoma, Norman, OK 73019, USA; huidou2015@gmail.com (W.D.); saikatchembiol@gmail.com (S.H.); oucmhy@gmail.com (H.M.); vickyanderson@ou.edu (V.M.A.); 5 Department of Chemistry, Oregon State University, Corvallis, OR 97331, USA; rd.overacker@gmail.com (R.D.O.); sandra.loesgen@whitney.ufl.edu (S.L.); 6 Whitney Laboratory for Marine Bioscience, Department of Chemistry, University of Florida, St. Augustine, FL 32080, USA

**Keywords:** natural products, Ewing sarcoma, drug discovery, fungal secondary metabolites, soil microbes

## Abstract

**Simple Summary:**

Ewing sarcoma is a cancer of the bone and soft tissues that affects children and adolescents. Unfortunately, only 20–30% of patients with metastatic Ewing sarcoma survive, necessitating the need to identify new, more effective therapies. We screened natural product extracts from plants and fungal cultures to identify compounds with selective cytotoxic activity against Ewing sarcoma cells, which led to the identification of altertoxin II as a compound with highly selective activity against Ewing sarcoma cells. Mechanism of action studies showed that altertoxin II selectively induces DNA damage in Ewing sarcoma cells, but does not bind to DNA. Additionally, we found that altertoxin II has antitumor activity in a mouse model of Ewing sarcoma, suggesting it will be useful as a lead compound to help identify new molecular targets for the development of new Ewing-sarcoma-specific therapies.

**Abstract:**

A screening program designed to identify natural products with selective cytotoxic effects against cell lines representing different types of pediatric solid tumors led to the identification of altertoxin II as a highly potent and selective cytotoxin against Ewing sarcoma cell lines. Altertoxin II, but not the related compounds altertoxin I and alteichin, was highly effective against every Ewing sarcoma cell line tested, with an average 25-fold selectivity for these cells as compared to cells representing other pediatric and adult cancers. Mechanism of action studies revealed that altertoxin II causes DNA double-strand breaks, a rapid DNA damage response, and cell cycle accumulation in the S phase. Our studies also demonstrate that the potent effects of altertoxin II are partially dependent on the progression through the cell cycle, because the G_1_ arrest initiated by a CDK4/6 inhibitor decreased antiproliferative potency more than 10 times. Importantly, the cell-type-selective DNA-damaging effects of altertoxin II in Ewing sarcoma cells occur independently of its ability to bind directly to DNA. Ultimately, we found that altertoxin II has a dose-dependent in vivo antitumor efficacy against a Ewing sarcoma xenograft, suggesting that it has potential as a therapeutic drug lead and will be useful to identify novel targets for Ewing-sarcoma-specific therapies.

## 1. Introduction

Ewing sarcoma (ES) is an aggressive bone and soft tissue cancer affecting children, adolescents, and young adults. This disease is most often caused by a chromosomal translocation leading to the expression of an abnormal fusion protein that is commonly designated as EWS-FLI1 [1,2]. While the prognosis for many patients is good, with 70–80% survival for localized cancers, only 20–30% of patients with metastatic or recurrent ES survive [2,3]. There is a clear need for new effective therapies to help treat these recalcitrant cases of ES and provide disease-specific therapies that cause fewer acute and chronic side effects. Based on the knowledge that ES tumors are caused by the expression of the EWS-FLI1 fusion protein, the possibility exists for the identification of targeted fusion-protein-dependent therapies that could achieve high levels of efficacy against ES tumors while avoiding toxicity to other tissues that do not express the abnormal fusion protein. To this end, a variety of EWS-FLI1-targeting approaches have been attempted, including the inhibition of EWS-FLI1 expression, the inhibition of the transcriptional activity of EWS-FLI1, and the repression of EWS-FLI1 downstream targets [3]. Unfortunately, the identification of a selective inhibitor of EWS-FLI1 has not yet been successful, in part because EWS-FLI1 is a highly disordered protein [4].

A screening program at the National Cancer Institute evaluated natural products from the Molecular Targets Laboratory at NCI (Frederick) for small molecules that inhibit EWS-FLI1 transcription. Their screen identified multiple natural products, including mithramycin [5], trabectedin (E-743) [6], and englerin A [7]. Mithramycin advanced to clinical trials in ES but failed because the serum levels necessary for the inhibition of transcription were not obtainable [8]. While trabectedin had promising effects in a Phase I clinical trial, the Phase II trial failed to show activity, with only one of 10 patients responding [9]. A new clinical trial of trabectedin in combination with irinotecan (SARC037) is currently enrolling patients (Clinicaltrials.gov (accessed on 1 December 2021)). 

Based in part on the success of the NCI in identifying multiple natural products with activity against ES, we investigated fungal-derived metabolites from the extensive natural product library at the University of Oklahoma, which contains >76,000 samples prepared from taxonomically diverse fungi, for compounds with selective cytotoxic activities against pediatric cancer cell lines. This approach has been effective in identifying multiple leads against subtypes of triple-negative breast cancer [10,11,12,13,14,15]. This unbiased, mechanism-blind screening program also led to the identification of compounds with selective cytotoxic activities against pediatric solid cancer cells lines, including ES [16,17]. Herein, we describe the identification of altertoxin II (ATXII) as a highly potent and selective cytotoxin against ES cells in vitro that also has antitumor activity in vivo against a murine xenograft model of ES.

## 2. Materials and Methods

### 2.1. General Experimental Procedures

Sulforhodamine B salt, Trizma, Dulbecco’s phosphate-buffered saline (DPBS), phenylmethanesulfonyl fluoride (PMSF), dimethyl sulfoxide (DMSO), crystal violet, and Kolliphor^®^ EL were purchased from Sigma-Aldrich (St. Louis, MO, USA). Acetic acid was purchased from Thermo Fisher Scientific (Waltham, MA, USA). Abemaciclib-mesylate was provided by Dr. Peter Houghton. The compounds used for in vitro cell treatments were dissolved in DMSO and stored at −20 °C. ATXII for in vivo studies was diluted in 50:50 DMSO:Kolliphor^®^ EL, stored at −20 °C, and diluted 1:10 in DPBS immediately prior to use. LCMS analyses were performed on a Shimadzu UFLC system with a quadrupole mass spectrometer using a Phenomenex Kinetex C18 column (3.0 mm × 75 mm, 2.6 μm) and a CH_3_CN-H_2_O (0.1% HCOOH) gradient solvent system. NMR spectra were obtained on a Varian a spectrometer (500 MHz for ^1^H and 125 MHz for ^13^C) using acetone-*d*_6_ (Aldrich) as the solvent. HPLC was performed on a Waters System equipped with a 1525 binary HPLC pump coupled to a 2998 PDA detector with a Phenomenex C18 column (21.2 × 250 mm or 10 × 250 mm, 5 μm).

### 2.2. Purification of Secondary Metabolites Altertoxin II, Altertoxin I, and Alteichin

The fungal isolate was identified as an *Alternaria* sp. (isolate code: SC5920 TV8-1) based on its ribosomal internal transcribed spacer (ITS) sequence data (GenBank accession number MW013191). The soil sample from which it was derived was obtained from South Carolina, USA, through the University of Oklahoma-s Citizen Science Soil Collection. This isolate was grown in three large mycobags (Unicorn Bags, Plano, TX, USA) charged with monolayers of Cheerios breakfast cereal supplemented with a 0.3% sucrose solution with 0.005% chloramphenicol. After four weeks, the contents of the bags were combined, homogenized, and extracted with EtOAc. The EtOAc extract (29.0 g) was subjected to silica gel vacuum liquid chromatography with elution steps of 1:1 hexanes-DCM, DCM, 10:1 DCM-MeOH, and MeOH, yielding four fractions. The 10:1 DCM/MeOH fraction (11.5 g) was then subjected to HP20SS vacuum liquid chromatography and elution performed using a step gradient of MeOH (30%, 50%, 70%, 90%, 100%) in water followed by 1:1 DCM-MeOH, yielding a total of six fractions. The bioassay analysis of the resulting fractions indicated that the selective cytotoxicity was limited to the 90% and 100% MeOH fractions. These two fractions were subjected to further bioassay-guided purification using preparative C18 HPLC (250 mm × 21.2 mm, 5 μm) with a MeOH-H_2_O gradient (30:70 to 100:0), followed by isocratic semi-preparative C18 HPLC (250 mm × 10 mm, 5 μm) with MeCN-H2O (50:80) containing 0.1% formic acid to yield altertoxin II (84.0 mg) and its structural analogues altertoxin I (8.0 mg) and alteichin (54.7 mg).

### 2.3. Phylogenetic Analysis of Citizen Science Alternaria

ITS sequences were generated from cell lysate using ITS1 (5′-TCCGTAGGTGAACCTGCGG-3′) and ITS4 (5′-TCCTCCGCTTATTGATATGC-3′). Amplification was performed using a LightCycle 480 II (Roche) using the following conditions: 1 cycle of denaturation at 94 °C for 2 min followed by 40 cycles of denaturation at 94 °C for 1 min, annealing at 50 °C for 1 min, and extension at 72 °C for 1 min. The samples were processed with Sanger sequencing by GENEWIZ. A cohort of 198 *Alternaria* sequences were then aligned using clustalW in Mega, and within group distances were generated using the Kimura2+G algorithm. A neighbor joining tree was constructed with 500 bootstraps using the same algorithm. 

### 2.4. Preparation of Alternaria Isolates for Metabolomic Analysis

Isolates indicated as belonging to *Alternaria* (based on BLAST comparisons to ITS data contained in the NCBI database) were cultured in duplicate on Cheerios breakfast cereal supplemented with a 0.3% sucrose solution spiked with 0.005% chloramphenicol. The cultures were extracted twice using a Tecan Freedom EVO^®^ robot. For the extraction process, a 3 mL aliquot of ethyl acetate was added to each culture tube followed by 3 mL of water. After 4 h, the ethyl acetate layer was transferred to a deep-well 96 well-plate. To increase the recovery rates of organic metabolites, a second 3 mL aliquot of ethyl acetate was added to each culture tube, which was removed after 2 h, and the ethyl acetate samples were combined. The organic solvent was removed under vacuum and the samples were stored at −20 °C for analysis.

### 2.5. LCMS Detection of Altertoxin II

The ethyl acetate soluble material from each fungus was suspended in 135 µL of 90:10 methanol that had been spiked with 0.5 μM sulfadimethoxine (internal quality control standard). LCMS analyses were conducted on a Thermo Fisher Scientific Vanquish Flex Binary LC system fitted with a C18 column (Kinetex, 50 × 2.1 mm, 1.7 µM, 100 Å, Phenomenex, Torrance, CA, USA) as an interface to a Thermo Fisher Q Exactive Plus hybrid quadrupole-orbitrap mass spectrometer. Sample elution was performed using a gradient system with increasing amounts of acetonitrile in H_2_O (treated with 0.1% formic acid). For the gradient, conditions were held at 5% acetonitrile/H_2_O for 1.0 min, increased to 100% acetonitrile over 8.0 min, and held for 2.0 min. The autosampler was maintained at 10 °C, while the column compartment was held at 40 °C. The samples were analyzed in random order with injection volumes of 5.0 μL. Blanks and pooled quality control samples were run after every 12 samples, alternating between a methanol blank and a media blank.

High resolution MS data were acquired in the positive ion mode using a scan range of *m*/*z* 100–1500, with a resolution of 35,000 and 17,500 for MS1 and MS2, respectively. MS2 data were acquired in a data-dependent manner: 5 MS/MS scans were acquired of the most abundant ion during each cycle. Both MS1 and MS2′s maximum injection time was 100 ms. The AGC target was 1E6 and 5E5 for MS1 and MS2, respectively. The isolation window was *m*/*z* 2. The sheath gas and auxiliary gas flow rate was set at 35 L/min and 10 L/min, respectively, at 350 °C, whereas the sweep gas flow rate was 0 L/min. The capillary temperature was maintained at 320 °C, and the spray voltage was 3.8 kV. The S-lens RF level was set to 50 V. MS2 data were collected at the apex within a window of 2–8 s and used normalized collision energy that was increased from 20% to 30% to 40%. Dynamic exclusion was used to avoid resampling ions within 10 s. Unassigned charges were excluded from the analysis. 

The peak corresponding to altertoxin II was identified by the comparison of the retention time and the MS2 spectrum of the metabolite’s [M+H-H_2_O]^+^ ion to an authentic sample of the metabolite. Intensity data for compound altertoxin II were plotted against the isolate number in the R software package. Isolates were classified as either high or low producers of altertoxin II based on the intensity of this ion. Isolates were characterized as high altertoxin II producers when the intensity measurement was greater than 1 × 10^7^. This value was used, since it corresponded to roughly one order of magnitude less than the intensity of the altertoxin II ion in the *Alternaria* sp. isolate SC5920 TV8-1 (≈4.30 × 10^8^).

### 2.6. Cell Culture

RD-ES, SK-ES-1, A-673, D283, A204, SH-SY-5Y, SJCRH30, SK-OV-3, MDA-MB-453, MDA-MB-231, HCC1806, and HCC1937 cell lines were purchased from the American Type Culture Collection (Manassas, VA, USA). The CAL-51 cell line was purchased from Creative Bioarray (Shirley, New York, NY, USA). The BT-549 cell line was obtained from Lombardi Comprehensive Cancer Center, in Georgetown University (Washington, DC, USA), and validated by Promega (Fitchburg, WI, USA). The TC32 cell line was provided by Dr. Alexander Bishop (University of Texas’ Health Science Center at San Antonio (UTHSCSA)). Cell line identities were confirmed by DNA short tandem repeat analyses (Labcorp, Burlington, NC, USA). TC32-NR0B1 and TC32-CMV cell lines were provided by Dr. Patrick Grohar (Van Andel Research Institute, Grand Rapids, MI, USA). The SK-N-BE(2)-C cell line was provided by Dr. Alexander Pertsemlidis (UTHSCSA). The Hep293TT cell line was provided by Dr. Gail Tomlinson (UTHSCSA). The SK-OV-3-MDR-1-6/6 is a single-cell clone we isolated from the SK-OV-3/MDR-1 cell line provided by Dr. Susan Kane (Division of Molecular Medicine, Beckman Research Institute of the City of Hope, Duarte, CA, USA) and cultured as previously described [18,19]. SK-ES-1, SH-SY-5Y, MDA-MB-453, MDA-MB-231, and SK-N-BE2 cells were cultured in Improved Minimum Essential Medium (Gibco/Thermo Fisher Scientific, Waltham, MA, USA) containing 25 μg/mL gentamicin (Gibco) and 10% fetal bovine serum (FBS; GE Healthcare, Little Chalfont, UK). TC32-NR0B1, TC32-CMV, RD-ES, A-673, D283, A204, SJCRH30, Hep293TT, HCC1806, BT-549, HCC1937, and Cal-51 cells were cultured in a RPMI-1640 medium (Sigma-Aldrich) containing 50 μg/mL gentamicin and 10% FBS. SK-OV-3 and SK-OV-3-M6/6 cell lines were cultured in Basal Medium Eagle (Sigma-Aldrich) containing 50 μg/mL gentamicin and 10% FBS. The cells were maintained in humidified incubators at 37 °C with 5% CO_2_. All cell lines were initially expanded and frozen as stocks in liquid nitrogen. The cells were passaged for less than 3 months after revival.

### 2.7. Sulforhodamine B Assay

The antiproliferative and cytotoxic activities of ATXII were evaluated using the sulforhodamine B (SRB) assay as previously described [20]. The cells were plated into 96-well plates at predetermined densities ranging from 2500 to 6500 cells/well, depending on the cell proliferation rate, and allowed to adhere overnight. The cells were treated with ATXII for 48 h prior to assessing antiproliferative and cytotoxic activities. The cell density at the time of treatment (T_0_) was measured to evaluate cytotoxicity. Concentration–response curves were plotted, and the concentrations causing 50% inhibition of proliferation compared to vehicle control (GI_50_), total growth inhibition (TGI), and 50% cell death compared to T_0_ (LC_50_) were interpolated from nonlinear regressions of the data (Prism 6; GraphPad Software, La Jolla, CA, USA).

### 2.8. Colony Formation Assays

A-673 ES cells were seeded at a density of 500 cells/60 mm tissue culture dish, allowed to adhere overnight, then treated with DMSO (0.5%) or the indicated concentrations of ATXII. After 4 h of treatment, the cells were washed with DPBS, and a fresh growth medium added. After 14 days of colony formation, the cells were fixed and stained with 0.5% crystal violet in 10% methanol. The colonies were counted using the GeneSnap software (PerkinElmer). The data were analyzed by one-way ANOVA with Tukey’s post hoc test using Prism 6.

### 2.9. Whole-Cell Lysis and Immunoblotting

A-673, RD-ES, and SK-ES-1 cells were treated with DMSO (maximum 0.5%) or ATXII for the indicated time periods, harvested by scraping, and lysed in a cell extraction buffer (Thermo Fisher Scientific) containing a protease inhibitor cocktail, PMSF, and Na_3_VO_4_ (Sigma-Aldrich). The total protein concentrations were measured with a Pierce Coomassie Plus assay kit (Life Technologies), and 10 μg of total protein was separated by SDS-PAGE on NuPAGE Bis-Tris gels (Life Technologies). The proteins were transferred to PVDF membranes (EMD Millipore, Billerica, MA, USA) and probed overnight with antibodies for Phospho-S345-Chk1, Chk1, Phospho-T68-Chk2, Chk2, Phospho-S15-p53, p53 (Cell Signaling Technology, Danvers, MA, USA), β-actin (Sigma), or FLI1 (Abcam, Cambridge, MA, USA; ab15289) diluted in an Odyssey blocking buffer in TBST (LI-COR Biosciences, Lincoln, NE, USA). The membranes were incubated with appropriate IRDye 680 or IRDye 800 secondary antibodies (LI-COR Biosciences) diluted in an Odyssey blocking buffer in TBST + 0.01% SDS. Near-infrared fluorescence signals were captured on an Odyssey FC (LI-COR Biosciences).

### 2.10. Flow Cytometry

The cell cycle distribution was evaluated by flow cytometry utilizing propidium iodide staining. A-673, RD-ES, and SK-ES-1 cells were treated with DMSO or the indicated concentrations of ATXII for 18 h. The cells were then harvested by scraping, washed in DPBS, and stained with Krishan’s reagent [21]. The DNA content was measured using a Muse Cell Analyzer (EMD Millipore). The data were analyzed with FlowJo 10 (FlowJo LLC, Ashland, OR, USA).

### 2.11. RNA Sequencing

TC32 cells were treated in triplicate with 10 nM ATXII for the indicated amount of time, and total RNA was isolated using an RNeasy mini kit (Qiagen, Germantown, MD, USA). Approximately 500 ng of total RNA was used for the RNA-seq library preparation by following the Illumina TruSeq stranded mRNA sample preparation guide (Illumina, San Diego, CA, USA). RNA-seq libraries were subjected to quantification and a subsequent 50 bp single-read sequencing module with the Illumina HiSeq 3000 platform. After the sequencing run, demultiplexing with CASAVA (Illumina, San Diego, CA, USA) was employed to generate the FastQ file for each sample. An average of ~35 M reads were generated for each sample. All RNA-seq FastQ reads were aligned with the reference genome (UCSC human genome build hg19) using TopHat2 [22] default settings. The BAM files obtained after alignment were processed using HTSeq-count [23] to obtain the counts per gene in all samples. The R package DESeq [24] was used to normalize gene expression with the size factor method and to perform pairwise comparisons between groups to identify differentially expressed genes (DEGs). Genes with an FDR-adjusted *p*-value < 0.1 and at least a 2-fold change compared to control were considered significantly differentially expressed. Upon obtaining the differentially expressed genes from all pair-wise comparisons, we performed k-means clustering on the combined gene set using MATLAB (MathWorks). An additional quality control statistical analysis of outliers, intergroup variability, distribution levels, PCA, and hierarchical clustering analysis were performed to validate the experimental data. A pathway analysis was performed with Ingenuity Pathway Analysis (IPA, Qiagen). A gene set enrichment analysis (GSEA) was performed with the software package distributed by the Broad Institute [25]. 

### 2.12. High-Content Immunofluorescence Imaging

A-673 and SJCRH30 cells were plated into black 96-well cell carrier plates (PerkinElmer) at a density of 3500 cells/well and allowed to adhere overnight. The cells were treated in triplicate with DMSO or the indicated concentrations of ATXII for 6 or 24 h, then fixed with paraformaldehyde. After fixation, the cells were incubated in a blocking solution of 10% bovine calf serum in DPBS for 20 min at room temperature. The cells were then incubated in a primary antibody against γ-H2A.X (1:400; Cell Signaling Technology) diluted in 1% bovine serum albumin/0.3% Triton X-100 in DPBS, overnight, at 4 °C. The cells were subsequently washed with DPBS and incubated with an Alexafluor-594-conjugated secondary antibody (1:1000; Life Technologies) for 1 h at room temperature. The plates were washed with DPBS, and the nuclei stained with NucBlue live cell stain (Life Technologies) diluted in DPBS. Images were collected using an Operetta high-content imaging system (PerkinElmer) using a 20× long working distance objective and analyzed with the Columbus Image Data Storage and Analysis System (PerkinElmer). A minimum of three fields were collected per well, with all concentrations tested in triplicate.

### 2.13. Luciferase Reporter Assay

*NR0B1* promoter activity was assessed using a luciferase reporter assay as previously described by Grohar et al. [6]. TC32-NR0B1 and TC32-CMV cells were plated in white, opaque-bottom 384-well plates (PerkinElmer) at a density of 5000 cells/well in 27 μL growth medium and allowed to adhere overnight. The cells were then treated at the indicated concentrations with ATXII diluted in 3 μL DMSO/growth medium for 6 h. Luciferase reporter activity was measured by adding 30 μL of steadylite plus a reporter gene assay reagent and measuring the luminescence on a Pherastar FS multimode plate reader (BMG Labtech). The results represent *n* = 3 independent experiments, with all concentrations tested in triplicate.

### 2.14. LLAMAS Assay

An aliquot (400 μL) of DNA solution (in the experimental group) or control buffer (in the control group) and 200 μL MeOH were mixed first prior to the addition of 10 μL alertoxin solution. The samples were passed through the ultrafiltration membrane (100 kDa cutoff) by centrifugation at 5000× *g* at 10 °C after 30 min incubation at room temperature. The resulting filtrates were collected for a LC-PDA-MS/MS analysis. In the dissociation step, the retained DNA-ligand complex in the upper chambers of the microcentrifuge tubes was washed with 30% MeOH in H_2_O and subjected to centrifugation at 5000× *g*, 10 °C. After washing, the DNA-ligand complex was mixed with 600 μL of 95% MeOH in H_2_O with 1% formic acid in a new tube and then incubated with periodic vortexing at room temperature for 20 min. The solubilized contents were then transferred to microcentrifuge tubes outfitted with new ultrafiltration filters and centrifuged at 5000× *g* at 10 °C for 10 min. The filtrates were subjected to in vacuo solvent evaporation and suspended in 50 μL MeOH for the LC-MS/MS analysis.

### 2.15. BioLayer Interferometry

DNA binding events were detected and monitored in real time using a FortéBio Octet Red 96 Biolayer Interferometer (Molecular Devices, now Sartorius) [26]. DNA sequences d(5′-biotin-GATTTCAAGATATTAAGAAG-3′), d(5′-CTTCTTAATATCTTGAAATC-3′), d(5′-biotin-GTGCCTGGACCGCCCGACCT-3′), and d(5′-AGGTCGGGCGGTCCAGGCAC-3′) were purchased from Integrated DNA Technologies (IDT, Coralville, IA). Streptavidin biosensors and Kinetics Buffer (1× PBS, pH 7.4, 0.02% Tween-20, 0.1% albumin, and 0.05% sodium azide) were purchased directly from Molecular Devices (San Jose, CA, USA). Single-stranded DNA oligomers were annealed for 2 min at 94°C followed by cooling to room temperature over 1 h. Duplex DNA was then stored at −20 °C as a 20 µM solution in a TE buffer (10 mM Tris, 0.5 mM EDTA, 50 mM NaCl, pH 8) until use. Biotinylated, double-stranded DNA was immobilized on streptavidin (SA) sensor tips for 1600 s at 25 nM in a 1× Kinetics buffer. Compound testing was done sequentially at 125 µM in the 1× Kinetics buffer with 5% DMSO using baseline, association, and dissociation steps for 60, 1600, and 1600 s, respectively. Double reference subtraction was performed to eliminate the signal associated with atypical binding events by subtracting data obtained using a separate set of blank sensors with DNA load and a set of sensors without DNA load, both in buffer [27,28]. The data were aligned using baseline signals and the curves fitted with a 1:1 best-fit model in FortéBio’s data analysis software. 

### 2.16. Xenograft Studies in Nude Mice

Female athymic nude mice (Envigo, Indianapolis, IN) were injected s.c. with 2 × 10^6^ A-673 cells and suspended in 100 μL DPBS and 100 μL Matrigel^®^ (BD Biosciences, San Jose, CA, USA), bilaterally into each flank. Once the tumors reached a minimum volume of 150 mm^3^, the mice were assigned to three different groups (*n* = 8 or 10 tumors/group). One group of mice received doses of 20 mg/kg on days 1, 3, 5, 8, 10, and 12, and another group of mice received doses of 40 mg/kg on days 1, 3, and 5. The third group consisted of untreated control animals. ATXII was administered by i.p. injection in a vehicle of 5% DMSO + 5% Kolliphor EL in DPBS. The mice were weighed and examined daily for signs of toxicity. The tumors were measured twice per week using calipers, and the tumor volume (mm^3^) was calculated as length (mm) × width (mm) × height (mm). All mice were housed in an AAALAC-approved facility at UTHSCSA and given food and water ad libitum. All procedures were IACUC-approved.

## 3. Results

### 3.1. Bioassay-Guided Purification of Altertoxin II

**Figure 1:**
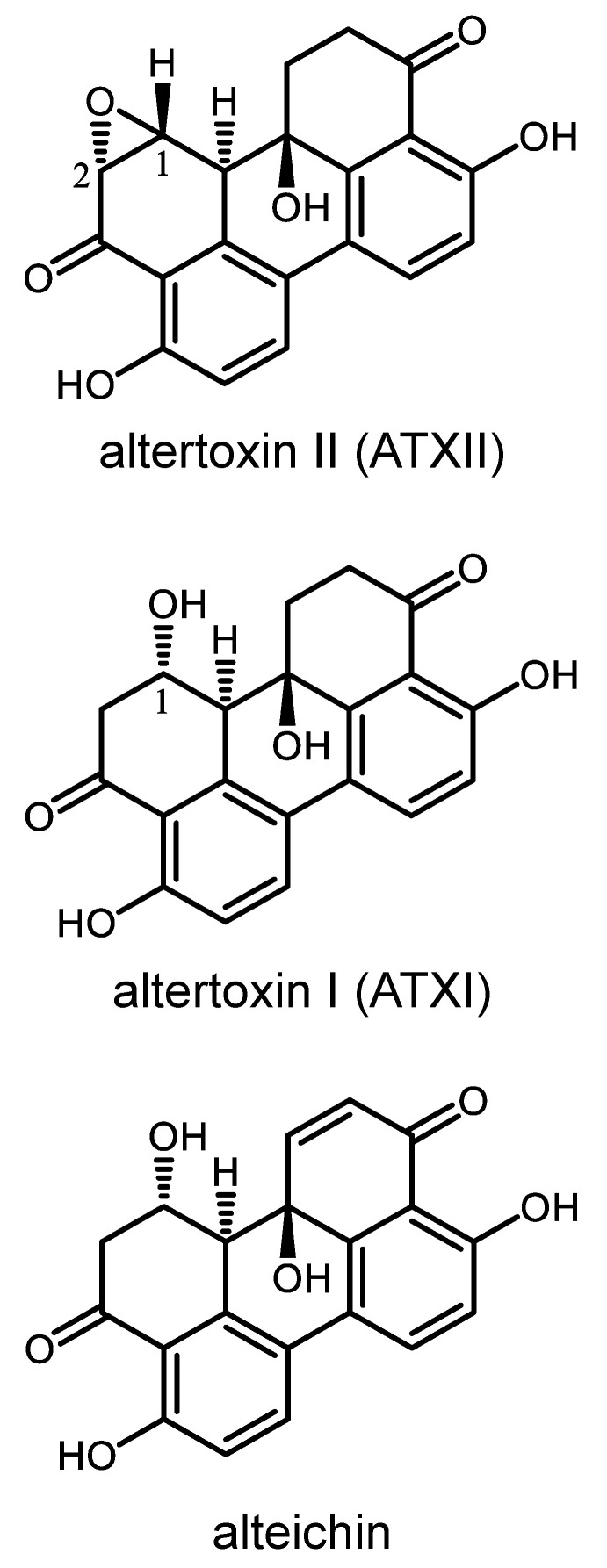
Chemical structures of ATXII and its analogues ATXI and alteichin purified from *Alternaria* sp. SC5920 TV8-1.

Crude extracts and fractions prepared from fungi isolated from a combination of Great-Lakes-derived sediments [29] and the University of Oklahoma Citizen Science Soil Collection [30] were evaluated for selective cytotoxic activity in cell lines modeling five different types of pediatric solid tumors: A-673 (Ewing sarcoma), SJCRH30 (rhabdomyosarcoma), SK-N-BE(2)-C (neuroblastoma), D283 (medulloblastoma) and Hep293TT (hepatoblastoma). A fraction obtained from a soil-derived *Alternaria* sp. isolate SC5920 TV8-1 exhibited selective cytotoxic effects against A-673 ES cells as compared to the other cell lines. Bioassay-guided fractionation of the active fungal sample yielded the perylene quinone-type metabolite altertoxin II (Figure 1). Concurrent with the bioassay-guided fractionation, LC-MS analysis was performed on the biologically active fraction, alerting us to the presence of two co-eluting metabolites. Based on their estimated molecular weights and photodiode array data, the metabolites were suspected of being putative analogues of ATXII. The purification and subsequent structure analysis of those compounds led to their identification as altertoxin I (ATXI) and alteichin (Figure 1). The dereplication of ATXII, ATXI and alteichin was carried out by comparing experimentally-derived data to published accounts of their mass spectrometry data and spectroscopic (i.e., 1D and 2D NMR, ECD, and optical rotation) properties [31,32,33,34,35]. 

**Figure 2:**
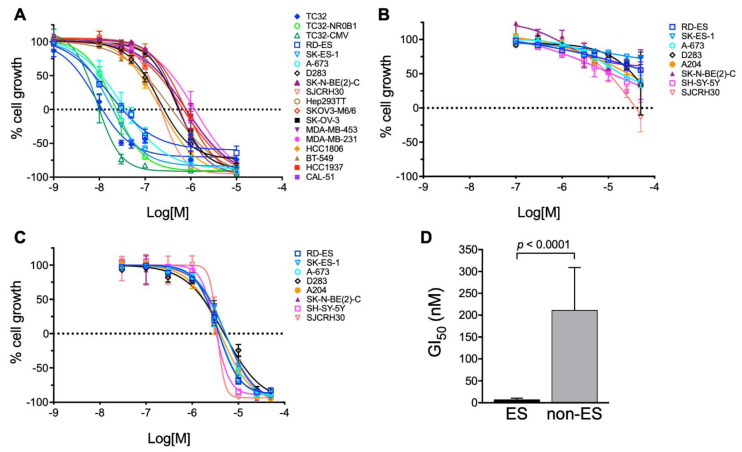
ATXII, but not altertoxin I or alteichin, has selective activity against Ewing sarcoma cells in vitro. (**A**) Sulforhodamine B (SRB) concentration-response curves for the inhibition of cell growth by ATXII, (**B**) altertoxin I and (**C**) alteichin. Results represent mean ± SE; *n* ≥ 3 independent experiments with each concentration tested in triplicate. (**D**) Comparison of GI_50_ (concentration resulting in 50% inhibition of cell growth relative to vehicle-treated control) for ATXII in EWS and non-EWS pediatric cancer cell lines. Results represent mean ± SD. Groups compared by two-tailed *t*-test.

ATXII, ATXI and alteichin were further evaluated in eight cell lines from our pediatric solid tumor panel representing ES (RD-ES, SK-ES-1, A-673) and non-ES (D283, A204, SK-N-BE(2)-C, SH-SY-5Y, and SJCRH30) cell lines. These cell lines were chosen because they represent four different types of pediatric solid tumors and demonstrated differential sensitivity to the fraction from which ATXII was isolated. The potent, ES-specific effects of ATXII were recognized to be a distinguishing feature of this metabolite, whereas ATXI and alteichin did not exhibit selective cytotoxic activity in ES cells compared to other pediatric cancer cell lines (Figure 2A–C). Considering that the only structural difference between the compounds is the presence of a 1,2-oxirane system versus a C-2 hydroxy group in ATXII and ATXI, respectively, we speculate that the epoxide group is critical for the ES-selective activity of ATXII.

**Figure 3:**
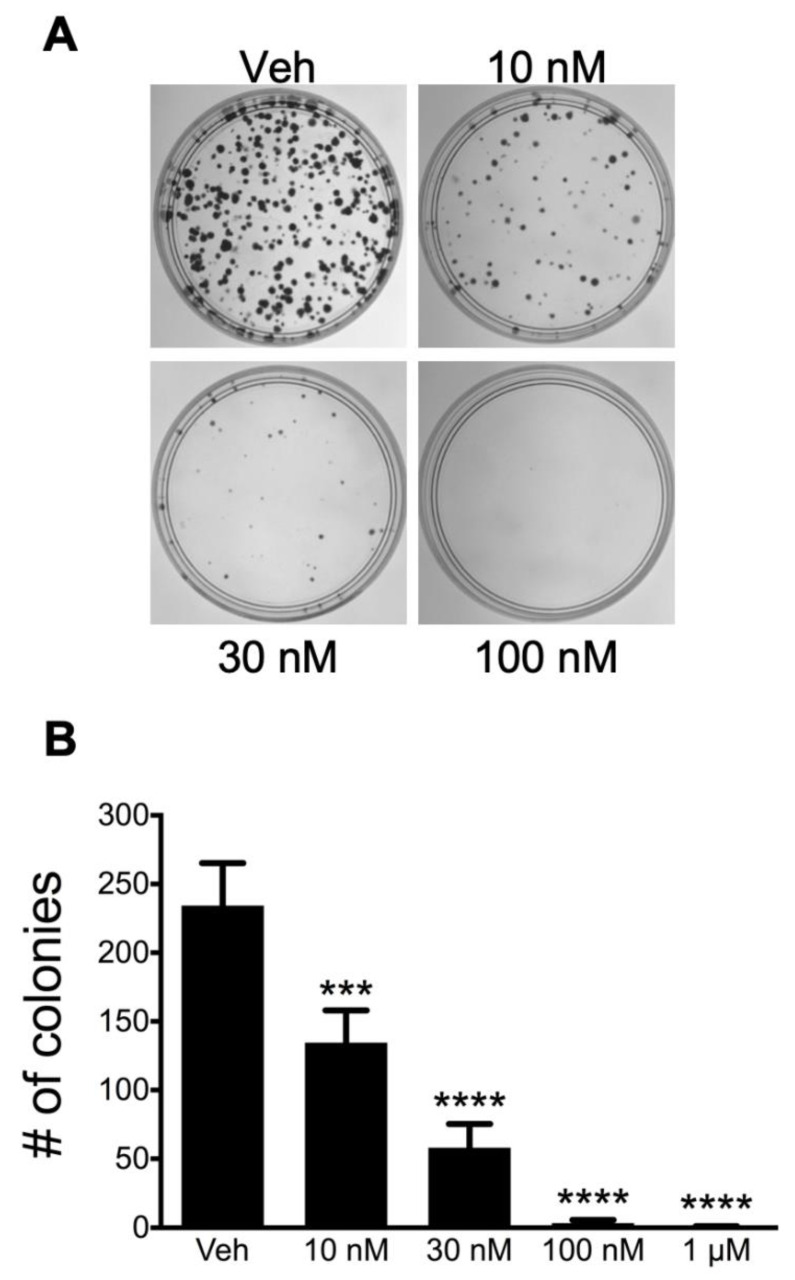
Altertoxin II has persistent activity in ES cells. (**A**) Inhibition of colony formation by ATXII after drug washout. A-673 cells were treated with ATXII for 4 h, the drug was washed out, and the cells were allowed to form colonies for 14 days. (**B**) Quantification of colony number after treatment with ATXII at the indicated concentration for 4 h followed by drug washout. *n* = 3; *** *p* ≤ 0.001, **** *p* ≤ 0.0001 compared to vehicle; one-way ANOVA with Tukey’s post-hoc test.

ATXII was next evaluated for antiproliferative and cytotoxic activities in a larger panel of six ES cell lines and 12 non-ES cell lines (four different pediatric solid tumor cell lines; two ovarian cancer cell lines, including SK-OV-3-MDR-1-6/6 cells that expresses P-glycoprotein; and six triple-negative breast cancer cell lines). ATXII showed a highly selective antiproliferative and cytotoxic effects in all of the ES cell lines with lower potency in each of the non-ES cells (Figure 2A; Table 1). The average concentration that caused a 50% inhibition of cell proliferation (GI_50_) was significantly different (*p* < 0.0001) between ES and non-ES cells (Figure 2D). The mean GI_50_ for ATXII was 8 ± 3 nM in the six ES cell lines and 200 ± 100 nM in non-ES pediatric cancer and adult cancer cell lines, indicating an average 25-fold selectivity for ES cells compared to non-ES cancer cells. In contrast, the GI_50_ values for ATXI and alteichin were 1.9 and 1.1-fold higher in ES cells compared to non-ES cells, respectively, indicating no selectivity for ES versus non-ES cells (Figure 2B,C). Similarly, the average concentration of ATXII that caused total growth inhibition (TGI) was significantly different (*p* < 0.0001) for ES and non-ES cells (Table 1). The mean TGI for ATXII was 20 ± 10 nM in the six ES cell lines and 600 ± 300 nM in non-ES cell lines, indicating an average of 30-fold selectivity for ES cells. The concentration of ATXII resulting in 50% cell death (LC_50_) was also significantly different (*p* = 0.0001) between ES and non-ES cells (Table 1), with a mean LC_50_ of 100 ± 100 nM in the six ES cell lines and 2000 ± 1000 nM in non-ES cell lines, demonstrating that ATXII has, on average, 20-fold cytotoxic selectivity for ES cells. ATXI and alteichin were not evaluated in this larger panel of cell lines because these compounds did not show any selectivity for ATXII when evaluated in the smaller panel of eight cell lines. The activity of ATXII was further assessed in A-673 ES cells by measuring its ability to inhibit colony formation. ATXII potently inhibited the colony formation of A-673 cells after a short, 4-h treatment followed by drug washout (Figure 3A,B). These data indicate that the effects of ATXII are highly persistent because these relatively short treatments with concentrations as low as 10 nM were sufficient to significantly inhibit the A-673 colony formation. 

### 3.2. ATXII Does Not Inhibit EWS-FLI1 Protein Expression or Transcriptional Activity

**Figure 4:**
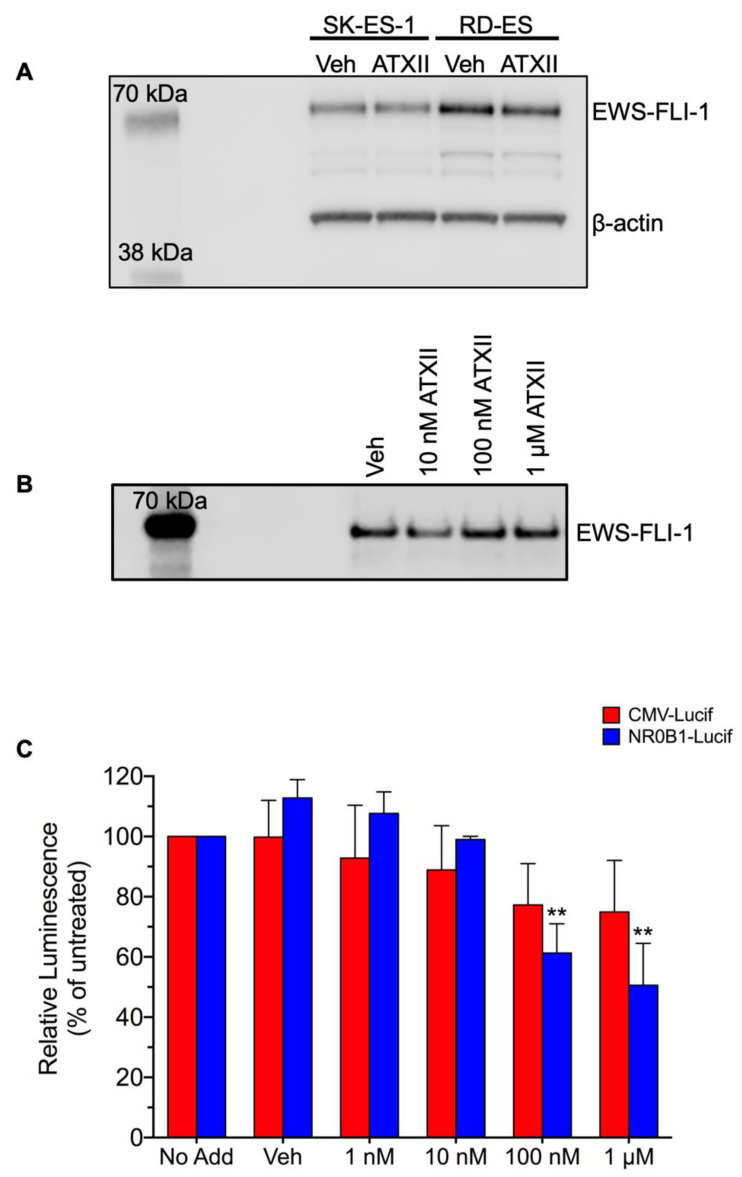
Effects of ATXII on EWS-FLI1 protein levels and transcriptional activity. (**A**) Immunoblotting for EWS-FLI1 (anti-FLI1) and β-actin in SK-ES-1 and RD-ES lysates following treatment with 100 nM ATXII for 8 h. (**B**) Immunoblotting for EWS-FLI1 (anti-FLI1) in A-673 lysates following treatment with a range of concentrations for 24 h. (**C**) Effects of ATXII on the promoter activity of the EWS-FLI1 target gene NR0B1. The cells were treated for 6 h with the indicated concentration of ATXII, and the activity was measured by a luciferase reporter assay. *n* = 3 independent experiments, with all concentrations tested in triplicate. ** *p* ≤ 0.01 compared to vehicle; one-way ANOVA with Tukey’s post-hoc test.

Due to the high degree of selectivity for ES cells, we assessed whether ATXII affects the abundance or transcriptional activity of EWS-FLI1, the oncogenic fusion protein that is the primary driver of ES. A relatively short time point (8 h) after treatment was evaluated to interrogate the early effects of ATXII on ES cells. The treatment of SK-ES-1 and RD-ES cells with 100 nM ATXII did not alter the EWS-FLI1 protein abundance, as assessed by immunoblotting (Figure 4A). Similarly, the treatment of A-673 cells with 10 nM–1 µM ATXII for 24 h did not affect the protein levels of EWS-FLI1 (Figure 4B). Together, these data indicate that ATXII does not alter the EWS-FLI1 protein abundance in ES cells and suggest that the depletion of EWS-FLI1 is not responsible for the selective cytotoxic effects of ATXII on ES cells. 

EWS-FLI1 acts as a transcription factor to globally modulate gene expression and drive an oncogenic phenotype in ES [1,36,37,38]. To determine if ATXII affects the transcriptional activity of EWS-FLI1, we evaluated the effects of ATXII on the promoter activity of NR0B1, a major downstream target of EWS-FLI1, using a luciferase reporter assay. TC32 ES cells stably expressing luciferase reporters under the control of either the *NR0B1* or cytomegalovirus (CMV) promoters were treated with ATXII for 6 h at concentrations ranging from 1 nM to 1 μM before evaluating the promoter activity (Figure 4C). The 1 and 10 nM concentrations of ATXII, the latter of which is sufficient to inhibit ES growth and colony formation (Figure 2A and Figure 3), had no effects on either the CMV or the *NR0B1* promoter activity as compared to vehicle-treated controls (Figure 4C). A small but statistically significant decrease in the *NR0B1* promoter activity was observed after treatment with higher concentrations, 100 nM or 1 μM ATXII, which was also accompanied by a decrease in the CMV promoter activity (Figure 4C). These data suggest that the decreased EWS-FLI transcriptional activity is not a major driver of ES growth inhibition and that the effects of higher concentrations of ATXII on the *NR0B1* promoter are more likely due to a generalized downregulation of transcription. 

### 3.3. ATXII Activates DNA Damage Response Pathways and Induces Double-Strand DNA Breaks in ES Cells

**Figure 5:**
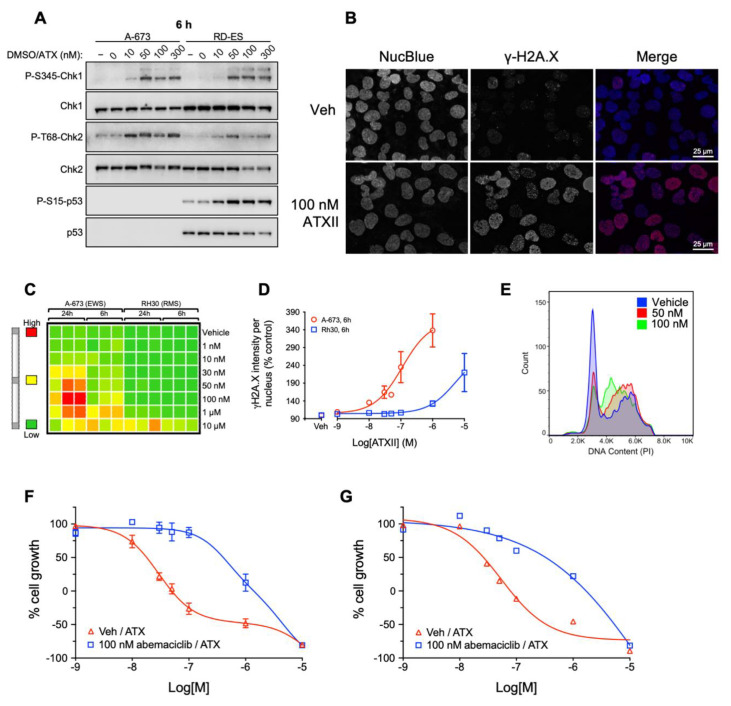
ATXII selectively induces DNA double-strand breaks, inhibits DNA synthesis, and is optimally potent in proliferating cells. (**A**) Immunoblotting for P-S345-Chk1, P-T68-Chk2, and P-S15-p53 in ES cell lines A-673 and RD-ES after a 6-h treatment with ATXII. (**B**) Indirect immunofluorescence microscopy of γ-H2A.X in A-673 cells after an 18-h treatment with ATXII. (**C**) Representative heatmap and (**D**) concentration-response curves for ATXII-induced γ-H2A.X accumulation in A-673 and Rh30 cells. The cells were treated in triplicate for 6 or 24 h with increasing concentrations of ATXII, and γ-H2A.X was measured by automated immunofluorescence imaging. Results represent mean ± SE; *n* = 2. (**E**) Analysis of cell cycle distribution by flow cytometry. A-673 cells were treated for 24 h with indicated concentrations of ATXII, and the DNA content was determined by PI staining of permeabilized cells. (**F**) Concentration-response curves for ATXII in RD-ES and (**G**) A-673 cells after pretreatment with the CDK4/6 inhibitor abemaciclib for 24 h. Results represent mean ± SE for *n* = 3 (RD-ES) or *n* = 1 (A-673) independent experiment(s) with each concentration tested in triplicate.

ATXII has been isolated from other fungi of the *Alternaria* genus, which are plant pathogens that cause the spoilage of food products, including grains and fruit [33,39,40]. Early studies showed that ATXII causes DNA damage in mammalian cells at concentrations of 250–750 nM, significantly higher than those where we observe ES-selective cytotoxicity [40,41]. Triggering such an effect would be highly relevant because previous studies have shown that ES cells are very sensitive to DNA damaging agents and exhibit high levels of DNA replication stress [42]. To determine if the induction of DNA damage is involved in the mechanism of action of ATXII in ES cells, the phosphorylation of checkpoint kinases 1 and 2 (Chk1 and Chk2) and p53 in A-673 and RD-ES ES cells was measured. The phosphorylation of Chk1 at S345, Chk2 at T68, and p53 at S15 is indicative of a cellular DNA damage response. The phosphorylation status of Chk1, Chk2, and p53 was assessed in ES cells after treatment with concentrations of ATXII ranging from 10 to 300 nM for 6 h to capture the acute effects of ATXII rather than secondary effects due to cell death (Figure 5A). In A-673 ES cells, the phosphorylation of Chk1 and Chk2 was observed at 6 h with 10 nM ATXII with a maximum phosphorylation of Chk1 occurring with 50 nM. The phosphorylation of Chk1 and Chk2 was also observed in the RD-ES cells, with the maximal phosphorylation obtained at 6 h with 50 nM ATXII (Figure 5A). An increased Chk2 phosphorylation was observed with concentrations as low as 10 nM. Interestingly, higher concentrations of ATXII (100 and 300 nM) resulted in lower levels of total Chk2 protein in RD-ES cells. A robust phosphorylation of p53 at S15 in RD-ES cells was observed at all the concentrations tested. The total levels of p53 decreased after treatment with 100 and 300 nM, although these may be secondary effects due to cell death at these higher concentrations. We did not detect total or P-S15-p53 in A-673 cells, which is consistent with previous studies showing that this cell line is p53-null [43].

ATXII and related compounds have been evaluated using the Ames assay, where they were shown to be mutagenic in *Salmonella typhimurium* strains [39]. Our results show that ATXII causes DNA damage in ES cells at concentrations much lower than those previously shown to induce general toxicity in cancer cells [40,41]. Consistently with these activities, other DNA-damaging drugs used to treat children with cancer were also positive in the Ames assay [44]. We evaluated the ES-selectivity of a panel of other DNA damage-inducing agents. including gemcitabine, etoposide, SN38 (active metabolite of irinotecan), melphalan, and the PARP1 inhibitor olaparib, but none of these agents show the degree of selectivity for ES cells that we see with ATXII (Supplementary Figure S1). These results suggest that ATXII induces DNA damage through a unique mechanism that cannot be repaired by ES cells. ES cells are understood to be highly sensitive to genotoxic stress, and they express low levels of key DNA repair genes, including *ATM* and *BRCA1* [45]. However, our results demonstrate that ATXII has a much greater selectivity for ES cells in vitro compared to other clinically relevant DNA-damaging agents.

EWS-FLI1 initiates DNA damage and transcriptional stress as measured by high levels of H2A.X phosphorylation at S139 (γH2A.X) and a slow replication fork progression [46]. Given the effects of the fusion protein and our finding that ATXII activates DNA damage response pathways in ES cells, studies were conducted to evaluate if ATXII induces the phosphorylation of H2A.X at S-139 (γ-H2A.X), a marker of DNA double-strand breaks. The ability of 100 nM ATXII to cause H2A.X phosphorylation was evaluated in A-673 cells following an 18-h treatment. The cells were stained with a DNA marker (NucBlue) and for γ-H2A.X with a phospho-specific antibody. The results show a robust increase in γ-H2A.X in the nuclei of ATXII-treated ES cells (Figure 5B). High-content immunofluorescence microscopy was performed in A-673 ES and SJCRH30 rhabdomyosarcoma (RMS) cells treated with 1 nM–10 µM ATXII for 6 or 24 h and the γ-H2A.X intensity per nuclei quantified for each condition. A heat map presentation of the results shows that γ-H2A.X was detected in A-673 ES cells at concentrations as low as 100 nM after 6 h and 30 nM after 24 h of treatment with ATXII (Figure 5C). In contrast, γ-H2A.X phosphorylation could only be detected in SJCRH30 RMS cells after treatment with 10 μM ATXII for 6 or 24 h. After 6 h of treatment, the EC_50_ (half maximal effective concentration) for ATXII-induced γ-H2A.X accumulation was 111 nM in A-673 cells and 5.3 μM in SJCRH30 cells, while after 24 h of treatment the EC_50_ was 67 nM in A-673 cells and 8 μM in SJCRH30 cells (Figure 5D). Interestingly, the concentration-response curves for ATXII-induced DNA damage (Figure 5D) and ATXII cytotoxicity (Figure 2A) in A-673 cells show that DNA damage and cell death occur over the same concentration range, with maximal effects achieved at 1 µM, suggesting that DNA damage is the primary driver of ATXII-induced cell death in ES cells.

A common consequence of DNA damage is cell cycle accumulation, either in the late G_1_- or S-phase, and thus the effects of ATXII on cell cycle distribution were evaluated in A-673 cells. The cells were treated with 50 or 100 nM ATXII for 24 h, resulting in a concentration-dependent accumulation of cells in the S-phase of the cell cycle (Figure 5E), suggesting that the ATXII-initiated DNA damage inhibits DNA synthesis, leading to a cell cycle checkpoint response and cell cycle arrest. Similar effects on cell cycle distribution were also observed in SK-ES-1 ES cells (Supplementary Figure S2).

Based on these results, we evaluated whether the potent cytotoxic effects of ATXII in ES cells require cell proliferation. RD-ES and A-673 cells were treated with the CDK4/6 inhibitor abemaciclib-mesylate, which caused the accumulation of cells in G_1_ at concentrations as low as 100 nM. Abemaciclib-mesylate did not initiate cytotoxicity at concentrations as high as 1 µM (Supplementary Figure S3). RD-ES and A-673 cells were first arrested in G_1_ by treatment with abemaciclib-mesylate for 24 h at 100 nM, a concentration that modestly inhibited growth but did not promote cytotoxicity on its own, followed by treatment with ATXII for 48 h. A pretreatment of RD-ES and A-673 cells with 100 nM abemaciclib-mesylate resulted in a rightward shift in the ATXII concentration-response curves (Figure 5F,G), increasing the GI_50_ 22.3-fold and 12.4-fold in RD-ES and A-673 cells, respectively, which were essentially equal to the potency in some non-ES cell lines. These data demonstrate that ES cells must be actively proliferating for ATXII to have maximal potency and further support the hypothesis that the potent cytotoxic effects of ATXII in ES cells are due to its ability to cause a selective induction of DNA damage in these cells at low concentrations.

To further probe the mechanisms of action of ATXII in ES cells, RNA sequencing (RNA-seq) was performed to assess changes in global gene expression in TC32 ES cells after 4, 8, and 12 h of treatment with 10 nM ATXII. TC32 cells were utilized for these studies in order to determine if ATXII has similar effects in multiple ES cell lines. We identified 43 genes that exhibit significant differences in expression (fold change > 2; adjusted *p*-value < 0.1) in ATXII-treated cells as compared to vehicle-treated cells (Supplementary Figure S4). Gene set enrichment analysis (GSEA) indicated significantly enriched gene sets related to DNA damage, including those that occur in response to ionizing radiation and cisplatin, as early as 4 h after ATXII treatment (Supplementary Figure S5). Collectively, these data strongly suggest that ATXII induces DNA damage in ES cells at concentrations as low as 10 nM. Interestingly, GSEA and gene ontology (GO) also indicate an enrichment in pathways related to type I interferon signaling and inflammatory response (Supplementary Figure S4), suggesting that these pathways might also play a role in the cytotoxic effects of ATXII. Induction of type I interferons can occur as a consequence of DNA damage due to ionizing radiation [47], and so the activation of these pathways is consistent with the induction of DNA damage by ATXII.

### 3.4. ATXII Does Not Directly Bind to DNA

**Figure 6:**
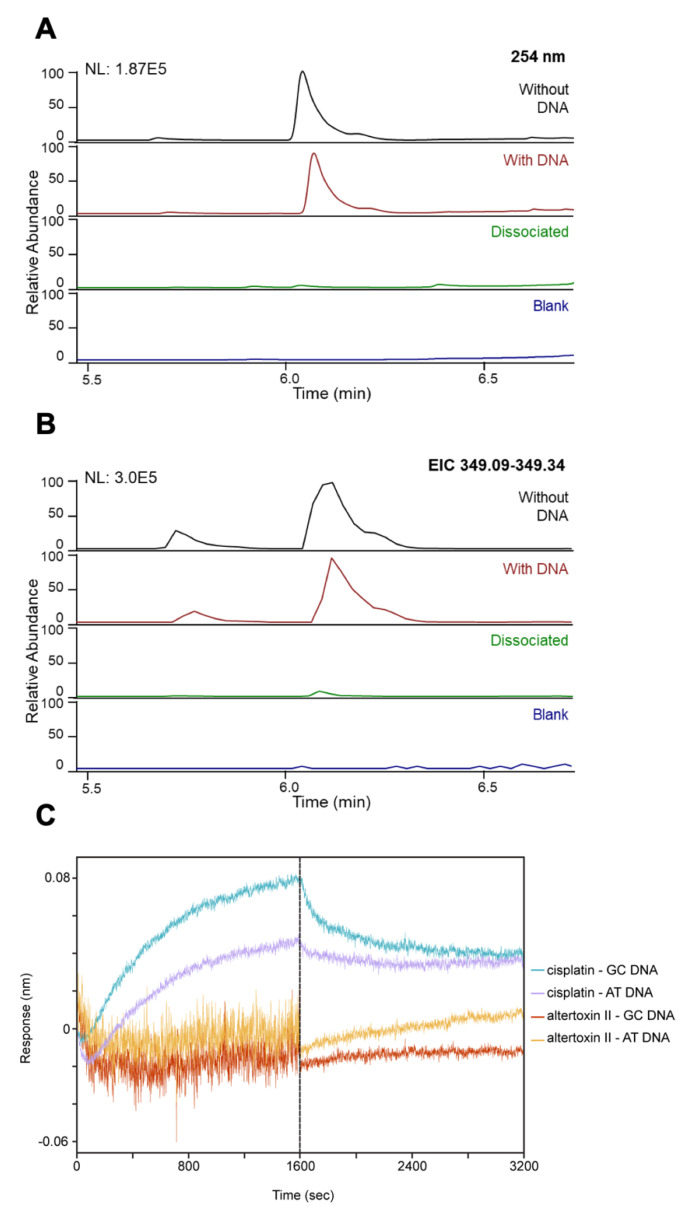
Altertoxin II does not directly bind to DNA. (**A**,**B**) DNA binding assay results of ATXII using LLAMAS. The UV chromatograms (λ 254 nm) and the extracted ion chromatogram (EIC) analysis revealed that ATXII does not show a recognizable DNA binding activity. (**C**) BioLayer interferometry binding sensorgrams for ATXII and cisplatin against GC- and AT-rich DNA (association step 0–1600 s and buffer dissociation step 1600–3200 s). Compounds evaluated at 125 μM against biotinylated, double-stranded DNA immobilized on streptavidin.

Based on our observations that ATXII induces DNA damage and activates DNA damage response pathways, we sought to determine if these effects were the result of the direct binding of ATXII to DNA. We recently developed a technique for identifying DNA-binding molecules in complex mixtures called lickety-split ligand-affinity-based molecular angling system (LLAMAS) [48]. Using this assay, no binding of ATXII to purified DNA was observed, suggesting that ATXII-induced DNA damage is not due to direct DNA binding (Figure 6A,B). To confirm these results, we performed biolayer interferometry assays to assess the binding of ATXII to GC and AT-rich DNA sequences. Whereas cisplatin showed detectable binding to DNA, we did not observe any binding of ATXII to the streptavidin-bound double-stranded DNA (Figure 6C). Collectively, these results indicate that ATXII does not directly bind to DNA and suggest that the ATXII-induced DNA damage is likely a consequence of the inhibition of a protein target, potentially one involved in DNA synthesis or damage repair. 

### 3.5. In Vivo Antitumor Efficacy of ATXII

**Figure 7:**
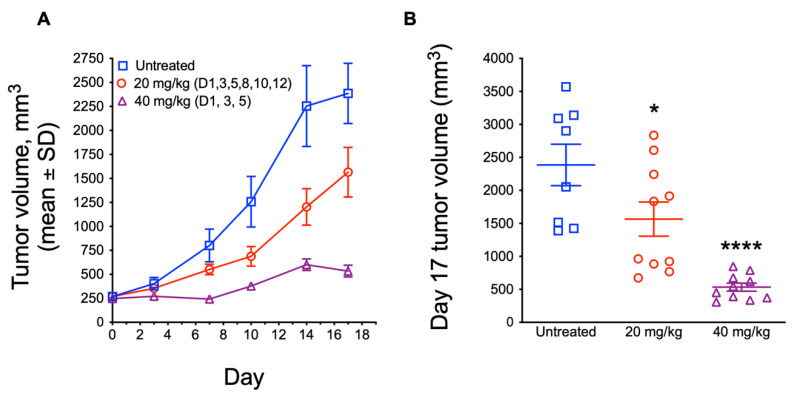
ATXII shows dose-dependent antitumor efficacy against an A-673 xenograft model. (**A**) Tumor growth curves for untreated control and ATXII-treated mice. The mice were injected i.p. with 20 mg/kg ATXII on days 1, 3, 5, 8, 10, and 12 or 40 mg/kg on days 1, 3, and 5. *n* = 8–10 tumors per group. (**B**) Comparison of final tumor volumes on day 17. * *p* ≤ 0.05, **** *p* ≤ 0.0001 compared to untreated control; one-way ANOVA with Tukey’s post-hoc test.

Our studies show that ATXII exhibits a highly selective cytotoxic activity against ES cells in vitro. To determine if ATXII has in vivo antitumor efficacy, we evaluated the effects of ATXII in an A-673 ES murine xenograft model. Mice bearing subcutaneous A-673 xenograft tumors of ~250 mm^3^ were treated with 20 mg/kg ATXII on days 1, 3, 5, 8, 10, and 12 or with 40 mg/kg ATXII on days 1, 3, and 5 for a total dose of 120 mg/kg in both cohorts. We observed a modest inhibition of tumor growth and minimal weight loss in the mice treated with the 20 mg/kg ATXII dose/schedule over the course of the 17-day trial (Figure 7A,B; Supplementary Figure S6). On day 17, the tumor volume was significantly smaller (*p* < 0.05) in this group compared to the control mice (Figure 7B). A less frequent dosing with 40 mg/kg ATXII on days 1, 3, and 5 resulted in a greater inhibition of tumor growth over the 17-day trial (Figure 7A,B). On day 17, tumors in mice in the 40 mg/kg ATXII group were significantly smaller than control tumors (*p* < 0.0001; Figure 7B). This dose and schedule caused more weight loss, although the mice recovered by the end of the trial (Supplementary Figure S6). Interestingly, the 40 mg/kg dose of ATXII produced long-lasting antitumor effects after the final dose was administered on day 5 of the trial, indicating a highly persistent antitumor activity. These results demonstrate that ATXII has antitumor efficacy against ES xenografts with an acceptable therapeutic window, and that ATXII is a possible lead molecule for the development of ES-specific therapies. 

## 4. Discussion

Unlike most adult cancers, that are caused by a lifetime of accumulated genetic changes, ES is caused by the expression of an aberrant transcription factor, EWS-FLI1, initiated most commonly by a t(11;22) (q24;q12) chromosomal translocation. Current therapies are effective for most children but are accompanied with long-term treatment-inducted morbidities [49,50]. New therapies that can improve survivors’ quality of life and the efficacy for patients with metastatic or recurrent disease are needed. The potential to target the unique vulnerabilities of ES caused by fusion protein expression suggest that targeted therapies for ES can be identified. Significant challenges remain, however, because EWS-FLI1 is a highly disordered transcription factor, making direct targeting unlikely. Additionally, the chimeric fusion protein causes both transcriptional activation and repression, which will be difficult to differentially regulate using a single therapeutic strategy to inhibit EWS-FLI1 transcription [2,51]. While ES is characterized as having a “quiet” mutational landscape, recent studies on the genetic vulnerabilities of ES cell lines show that they have complex dependencies, rivaling those of adult cancers [52]. Interestingly, these genetic dependences are different from the common vulnerabilities seen in adult cancers, highlighting the need to discover ES-specific therapies. 

Natural products have a long history of use as pharmaceuticals, and several natural products, including mithramycin, englarin A, and trabectedin were identified in a screen for inhibitors of EWS-FLI1 transcription [5,6,7]. The success of this screen suggests that the investigation of additional natural sources could be fruitful. We embarked on a discovery project using a high-throughput phenotypic screen of natural product extracts and fractions that is based on identifying mixtures containing constituents with selective cytotoxic activities against ES cells. A mechanism-blind phenotypic screen has the advantage of being unbiased towards the target and provides the opportunity to identify new unanticipated targets. The initial detection of an active fungal fraction, followed by bioassay-guided fractionation, identified ATXII as a highly selective cytotoxin in ES models. Our results confirm the continuing value of screening natural product libraries for compounds with selective activities against ES. 

The advantage of a differential sensitivity screen is that compounds with high selectivity can be identified from complex mixtures. This was held to be true in that ATXII has exquisite potency against every ES cell line that we tested as compared to all other childhood and adult solid tumor cell lines. In our decades of experience, this highly consistent degree of ES selectivity, greater than 20-fold, is noteworthy. While the cytotoxic effects of ATXII in cancer cell lines have been previously described, the potencies reported are in the range of the non-ES cell lines in our study, reinforcing that ES lines are uniquely sensitive to ATXII [40,41]. Mutagenic effects of ATXII in the Ames assay have been reported, but the mutagenic effects in mammalian cells also occur at concentrations higher than those that inhibit the proliferation and promote cytotoxicity in ES cell lines [39,40,41]. There is good reason to be cautious about the use of a mutagen in children and young adults, but it should be noted that the drugs used for the treatment of ES, including doxorubicin, cyclophosphamide, and melphalan [2], are also mutagenic in the Ames assay [44]. 

The structure–activity relationships among ATXII, ATXI, and alteichin demonstrate the critical importance of the epoxide moiety in the potency and ES selectivity of ATXII. Considering the bias that epoxide groups are indiscriminately reactive, we were nevertheless surprised when our studies showed that ATXII does not bind directly to DNA. Yet while ATXII does not bind to DNA, it causes a rapid DNA damage response at concentrations as low as 10 nM, specifically in ES cells. Interestingly, previous studies have demonstrated that ES cells exhibit high levels of DNA replication stress and are particularly susceptible to DNA damaging agents [42]. Our results are consistent with these findings. However, we demonstrate that many clinically relevant agents that induce DNA damage, including topoisomerase inhibitors and the poly (ADP-ribose) polymerase (PARP) inhibitor olaparib, do not show the same degree of selectivity for ES that we observe with ATXII, suggesting that ATXII induces a unique type of damage that is particularly difficult for ES cells to repair. Our findings also indicate that the cytotoxic effects of ATXII are partially dependent on cellular proliferation, suggesting the ability of ATXII to interfere with DNA replication.

The high levels of DNA replication stress are a defining feature of ES, although the direct molecular causes of this replication stress are not clear. This phenotype of elevated replication stress induces genomic instability and is thought to confer sensitivity to DNA damaging agents. Replication stress can be caused by a variety of factors, including the deregulation of nucleotide synthesis, the inhibition of DNA polymerase/helicase, and the accumulation of DNA–RNA hybrids(R-loops), among others [53,54]. Indeed, ES shows elevated levels of R-loops compared to other cancer types, and it is thought that these R-loops induce genomic instability and a sensitivity to DNA damaging agents [42]. Our results suggest that the high sensitivity of ES cells to ATXII results from an undefined yet selective induction of DNA damage. However, our data additionally demonstrate that ATXII does not directly bind to DNA, suggesting that it induces DNA damage through an indirect mechanism. ATXII might inhibit DNA repair through the direct inhibition of a protein involved in the cellular DNA damage response or inhibition of DNA replication, leading to an accumulation of stalled replication forks. Interestingly, we observed the accumulation of ES cells in the S phase after treatment with low concentrations of ATXII, and the effects of ATXII are at least partially dependent on cellular proliferation. These data are consistent with ATXII inhibiting DNA replication, which could increase the replication stress and lead to an accumulation of DNA damage. In the future, we plan to conduct additional binding studies with radiolabeled ATXII, which will be used to identify its direct molecular target(s). Additionally, a CRISPR-Cas9-mediated gene knockout screen will be employed to further define the mechanism of ATXII-induced DNA damage in ES cells [55]. Lastly, the development of an ATXII-resistant ES cell line, followed by whole-exome and RNA sequencing, would be useful in identifying both genetic and epigenetic mechanisms of ATXII resistance, and may provide further insight into its mechanisms of action. 

We demonstrate that altertoxin II has in vivo antitumor efficacy against an A-673 xenograft model of ES. It is likely that an increased antitumor efficacy will be observed by combining ATXII with other agents that act through a different mechanism of action. Specifically, ES is known to exhibit a “BRCAness” phenotype with deficiencies in homologous recombination [42], making these tumors particularly sensitive to PARP inhibitors. It is possible that ATXII will show increased efficacy in the combination with PARP inhibitors, or that a lower dose of ATXII may be utilized to minimize the general toxicity. Our future studies will be aimed at identifying optimal drug combinations with ATXII both in vitro and in vivo to treat ES tumors. 

## 5. Conclusions

We identified the fungal metabolite ATXII as a compound with highly selective cytotoxic activity against ES cells. Mechanism of action studies indicate that ATXII selectively induces DNA double-strand breaks and S-phase accumulation but does not directly bind to DNA. The high degree of selectivity for ES cells suggests that ATXII acts through a unique mechanism of action compared to other clinically relevant DNA-damaging agents. Overall, the efficacy of ATXII in an ES xenograft model, combined with its unique mechanistic effects, demonstrates that ATXII will be a valuable chemical probe for identifying ES-specific vulnerabilities and new drug targets for ES and as a potential therapeutic lead for this pediatric cancer. 

## Figures and Tables

**Table 1:** Sulforhodamine B assay potency measurements for ATXII. ES, Ewing sarcoma; RMS, rhabdomyosarcoma; Med, medulloblastoma; NB, neuroblastoma; HB, hepatoblastoma; OV, ovarian, BR, breast.

Cell Line	Type	GI_50_ (nM)	TGI (nM)	LC_50_ (nM)
RD-ES	ES	7.8	32	380
SK-ES-1	ES	7.6	20	58
A-673	ES	11	37	150
TC32	ES	4.0	12	48
TC32-NR0B1	ES	10	23	57
TC32-CMV	ES	5.0	10.0	20
SJCRH30	RMS	120	220	430
D283	Med	100	260	880
SK-N-BE(2)-C	NB	270	570	1200
Hep293TT	HB	240	540	1300
SK-OV-3	OV	250	550	1300
SK-OV-3-MDR1-6/6	OV	320	770	1900
MDA-MB-453	BR	230	790	2700
MDA-MB-231	BR	400	1200	3700
HCC1806	BR	80	220	650
BT-549	BR	100	380	1500
HCC1937	BR	200	680	2400
CAL-51	BR	230	810	2800

## Data Availability

RNA sequencing data will be made publicly available at the Gene Expression Omnibus (GEO) of the NCBI. All other data are available upon request.

## Supplementary Materials

The following are available online at https://www.mdpi.com/article/10.3390/cancers13246176/s1, Figure S1: Clinically used DNA-damaging agents do not show selectivity for Ewing sarcoma cells, Figure S2: Analysis of cell cycle distribution in SK-ES-1 cells by flow cytometry, Figure S3: Analysis of abemaciclib-induced cytotoxicity and cell cycle arrest in RD-ES cells, Figure S4: Heatmap of differentially expressed genes in TC32 cells following ATXII treatment, Figure S5: Gene set enrichment analysis of TC32 cells treated with ATXII, Figure S6: Mean percent change in mouse weights over the course of the trial.
