# Supplementary material for: Altertoxin II, a Highly Effective and Specific Compound against Ewing Sarcoma
Source: Cancers (Basel). 2021 Dec 7;13(24):6176. doi: 10.3390/cancers13246176 (PMC8699301; doi:10.3390/cancers13246176)
Supplement: Supplementary file 1 [file cancers-13-06176-s001.zip › cancers-1465156-supplementary.pdf]

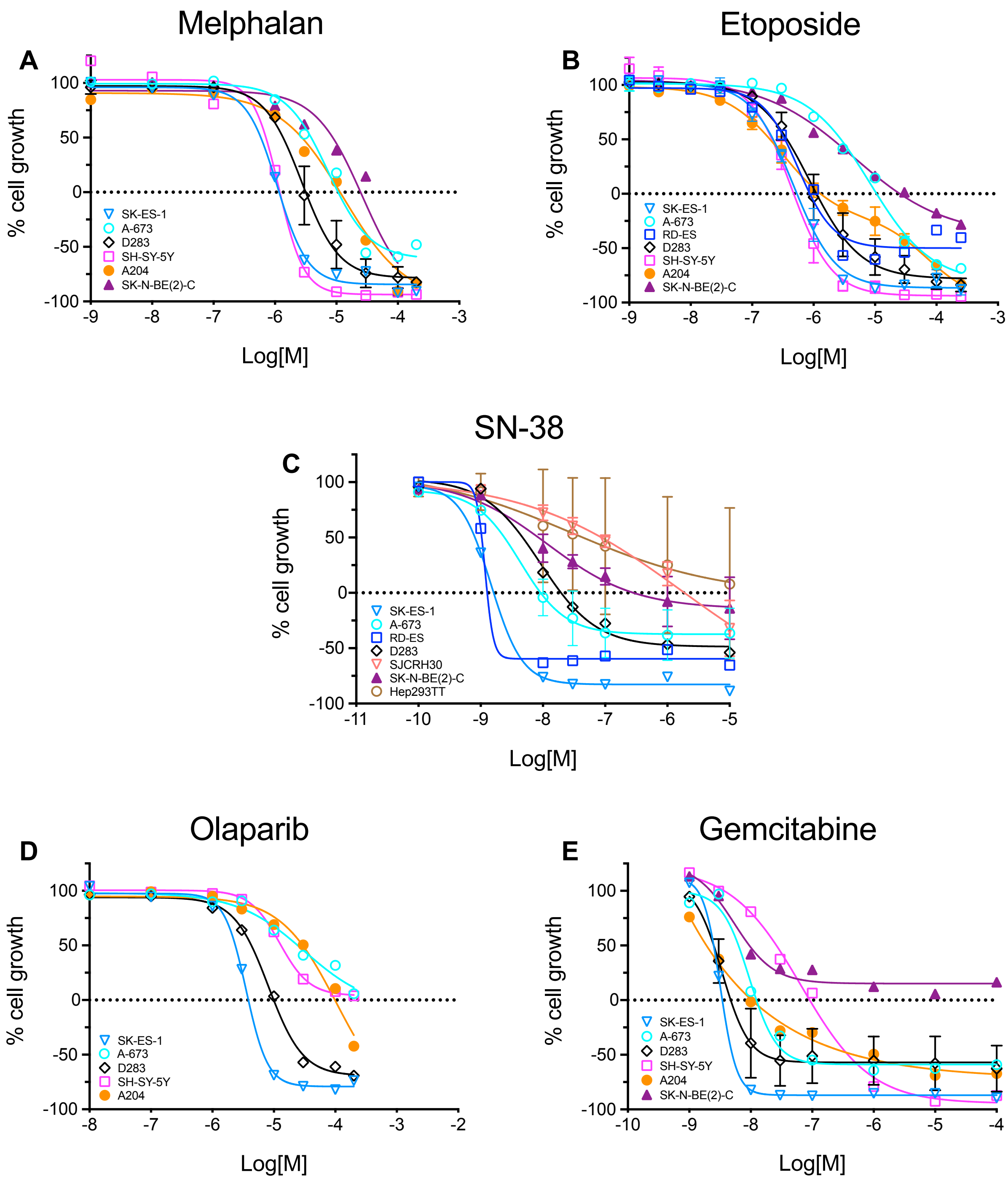

Figure S1. Clinically used DNA damaging agents do not show selectivity for Ewing sarcoma cells. Sulforhodamine B (SRB) concentration-response curves for inhibition of cell growth by (A) melphalan, (B) etoposide, (C) SN-38, (D) olaparib and (E) gemcitabine. Cells were treated with indicated compounds for 48 h prior to assessment of cell growth and viability. Results represent mean ± SE for n = 1-2 independent experiments with each concentration tested in triplicate.

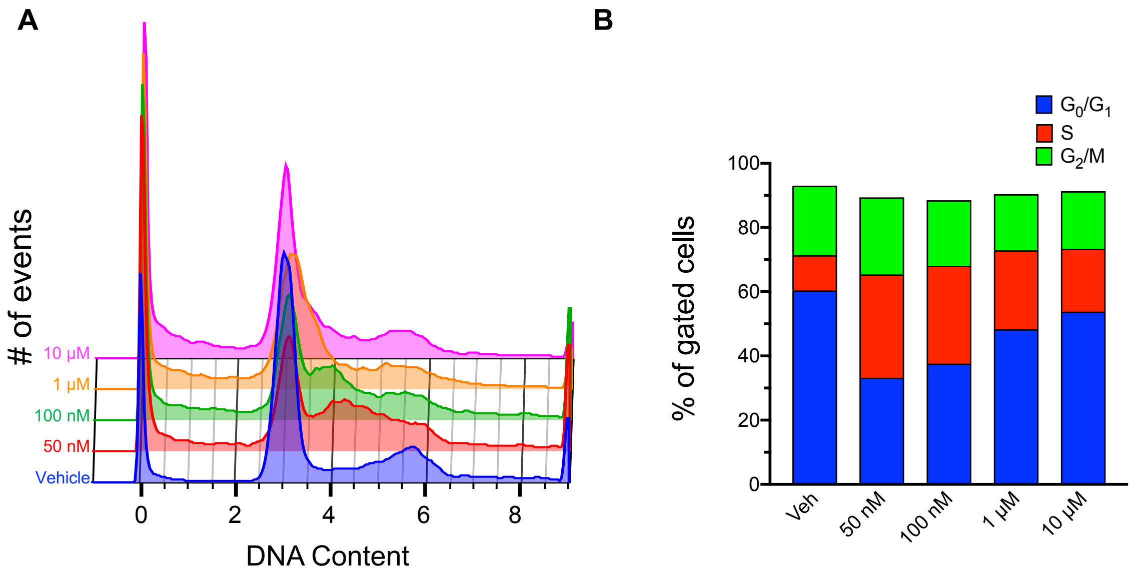

Figure S2. Analysis of cell cycle distribution in SK-ES-1 cells by flow cytometry. (A) Histogram of DNA content and (B) Quantification of percent gated cells in different cell cycle phases. Cells were treated for 18 h with indicated concentrations of ATXII and stained with Krishan's reagent.

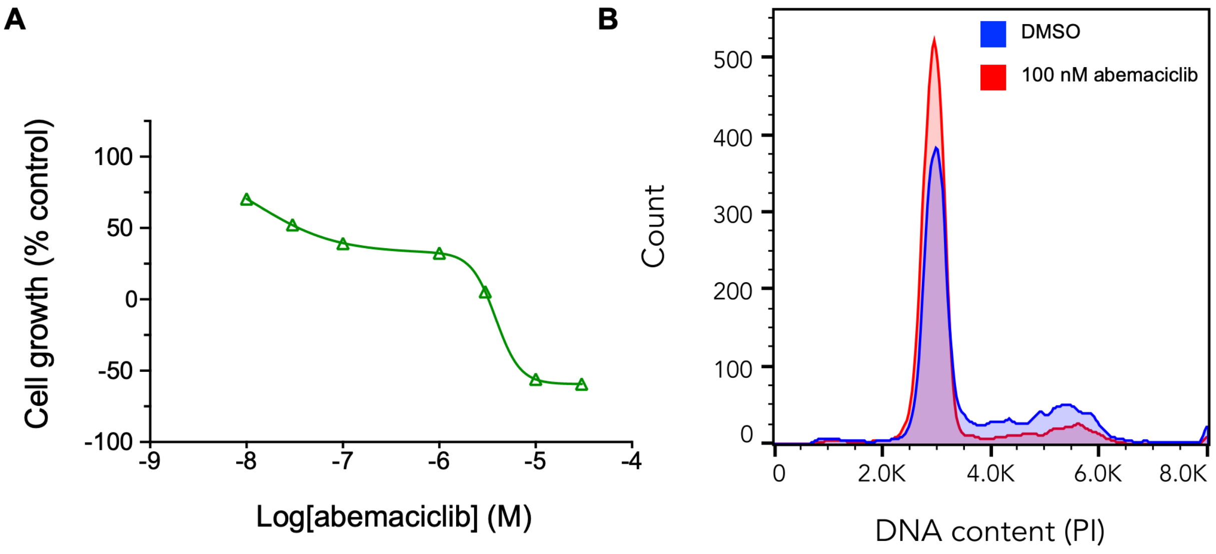

Figure S3 (A) Concentration-response curve for abemaciclib-mesylate in RD-ES cells after 72 h treatment. (B) Cell cycle profile for RD-ES cells after treatment with 100 nM abemaciclib-mesylate for 24 h.

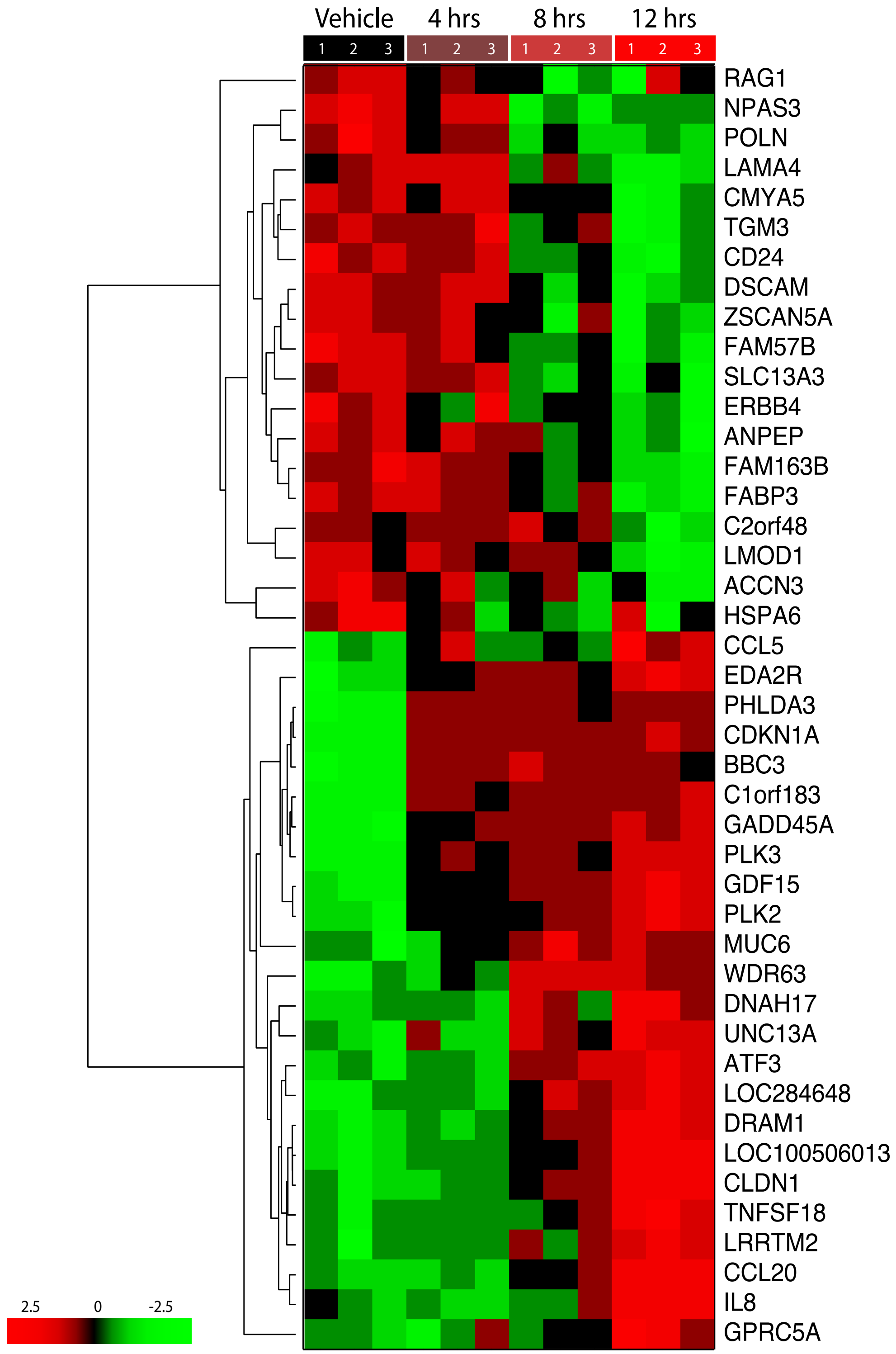

Figure S4. Heatmap of differentially expressed genes following treatment of TC32 cells with 10 nM ATXII for 4, 8 and 12 h, evaluated by RNA-sequencing. Cells were treated in triplicate for each of the indicated time points.

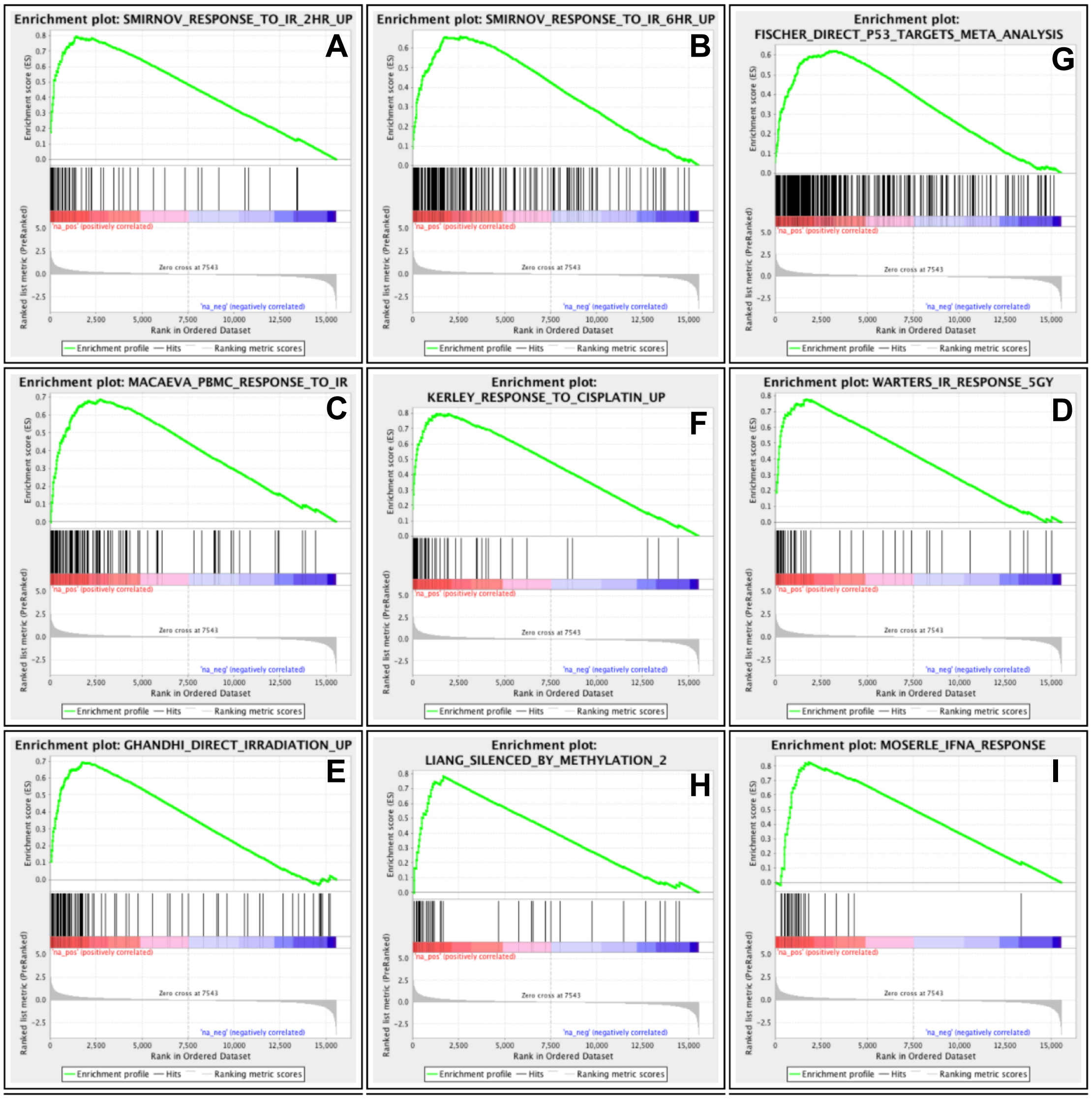

Figure S5. Gene set enrichment analysis (GSEA) enrichment plots showing enrichment of genes upregulated in response to ionizing radiation (A-E), cisplatin (F), decitabine (H) or interferon alpha (I) and p53 targets (G) in TC32 cells treated with 10 nM ATXII for 4, 8 or 12 h. RNA expression was evaluated by RNA-sequencing.

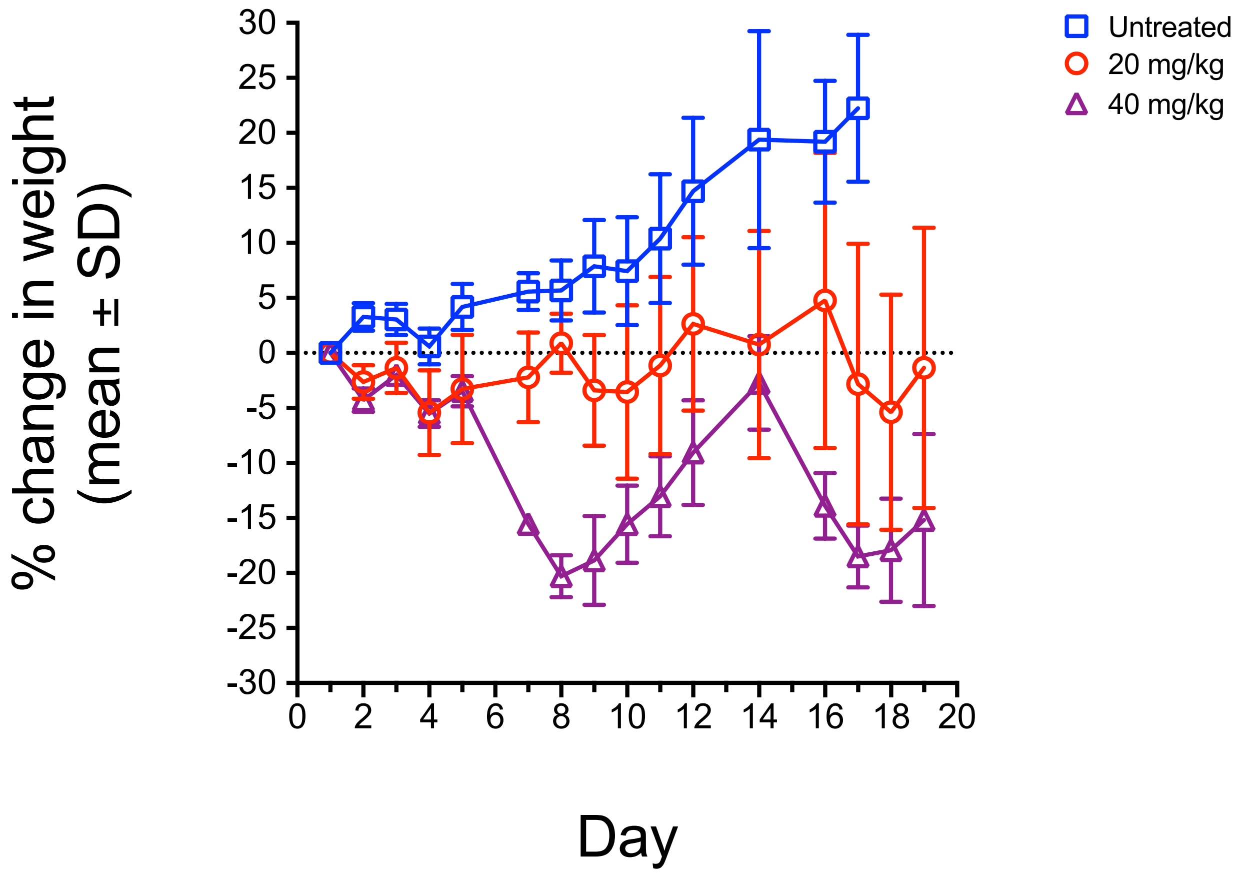

Figure S6. Mean percent change in mouse weights over course of trial. Mice were injected i.p. with 20 mg/kg ATXII on days 1, 3, 5, 8, 10 and 12 or 40 mg/kg on days 1, 3 and 5. n = 8-10 tumors per group
