# Supplementary material for: Altertoxin II, a Highly Effective and Specific Compound against Ewing Sarcoma
Source: Cancers (Basel). 2021 Dec 7;13(24):6176. doi: 10.3390/cancers13246176 (PMC8699301; doi:10.3390/cancers13246176)

Figure 4A – EWS-FLI-1 and β-actin (merged)

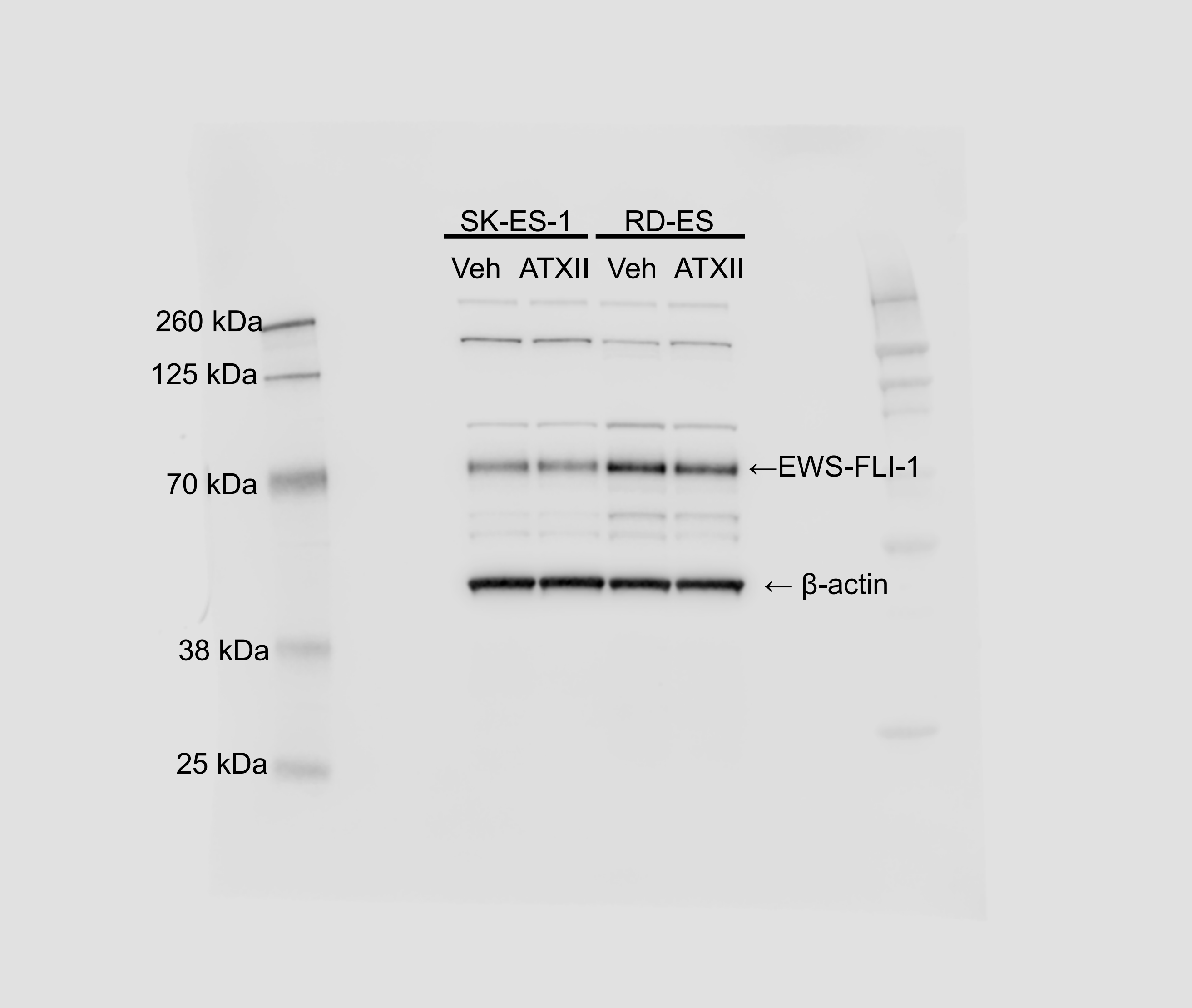

Figure 4A – EWS-FLI-1

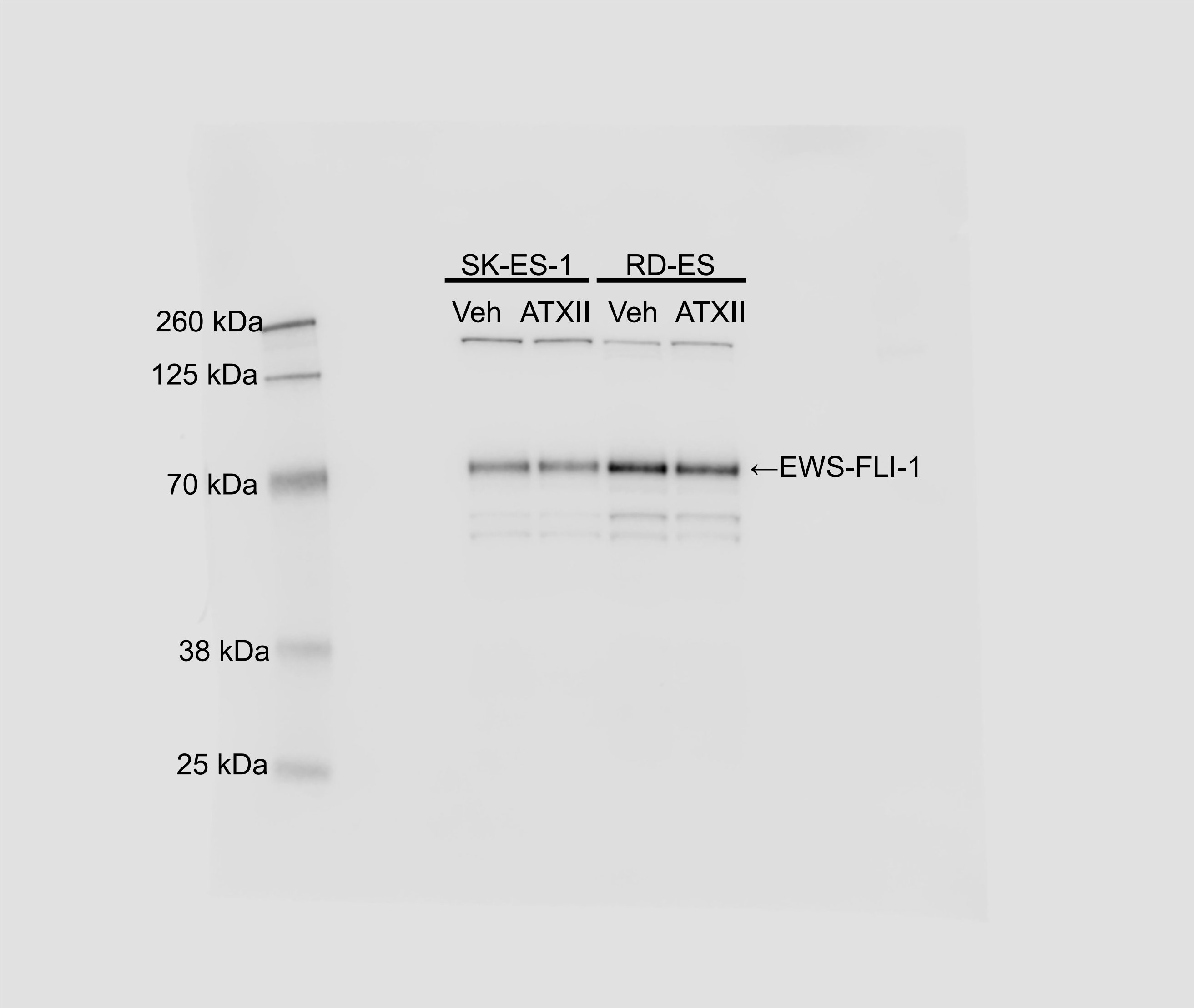

Figure 4A – β-actin

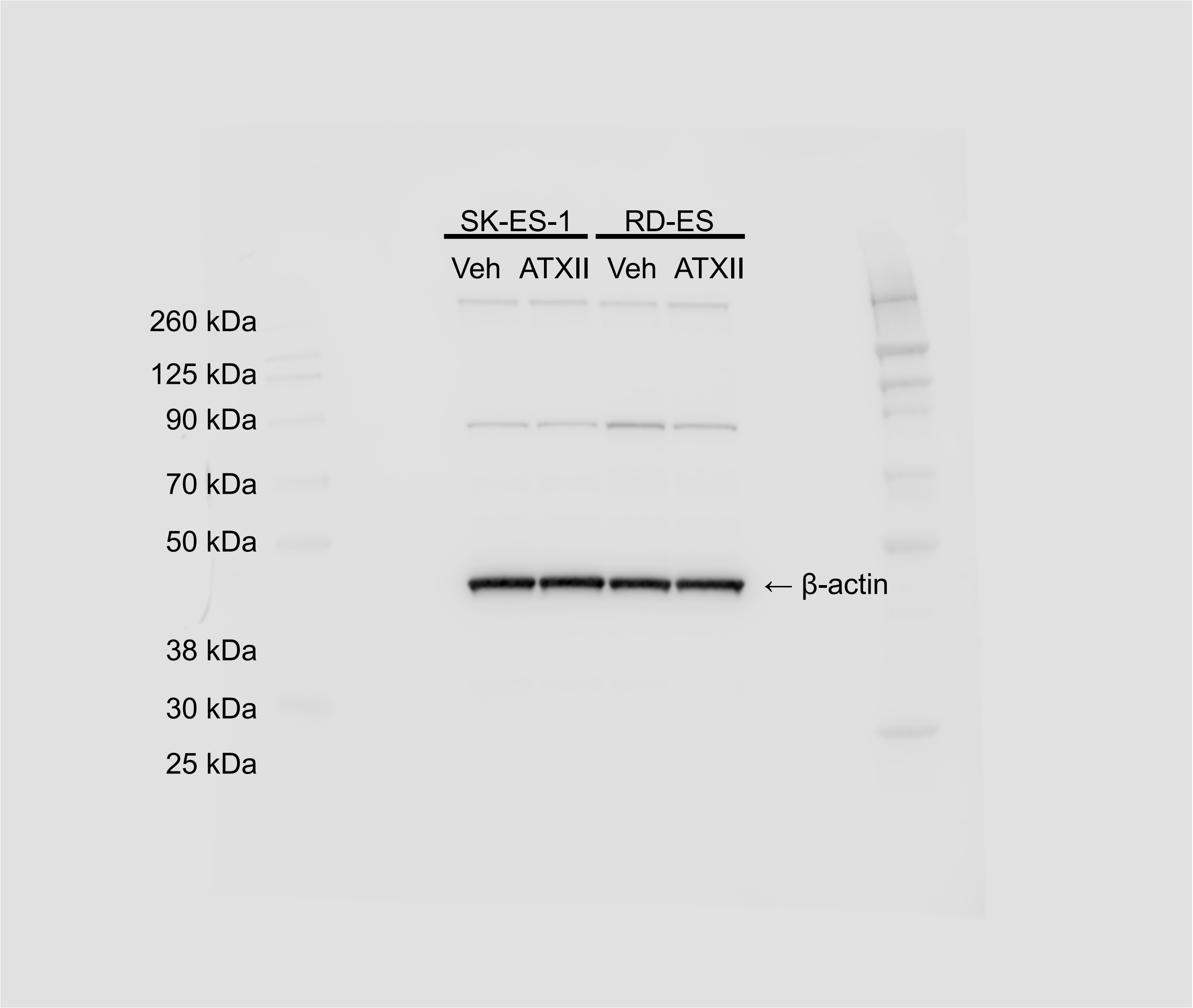

Figure 4B – EWS-FLI-1

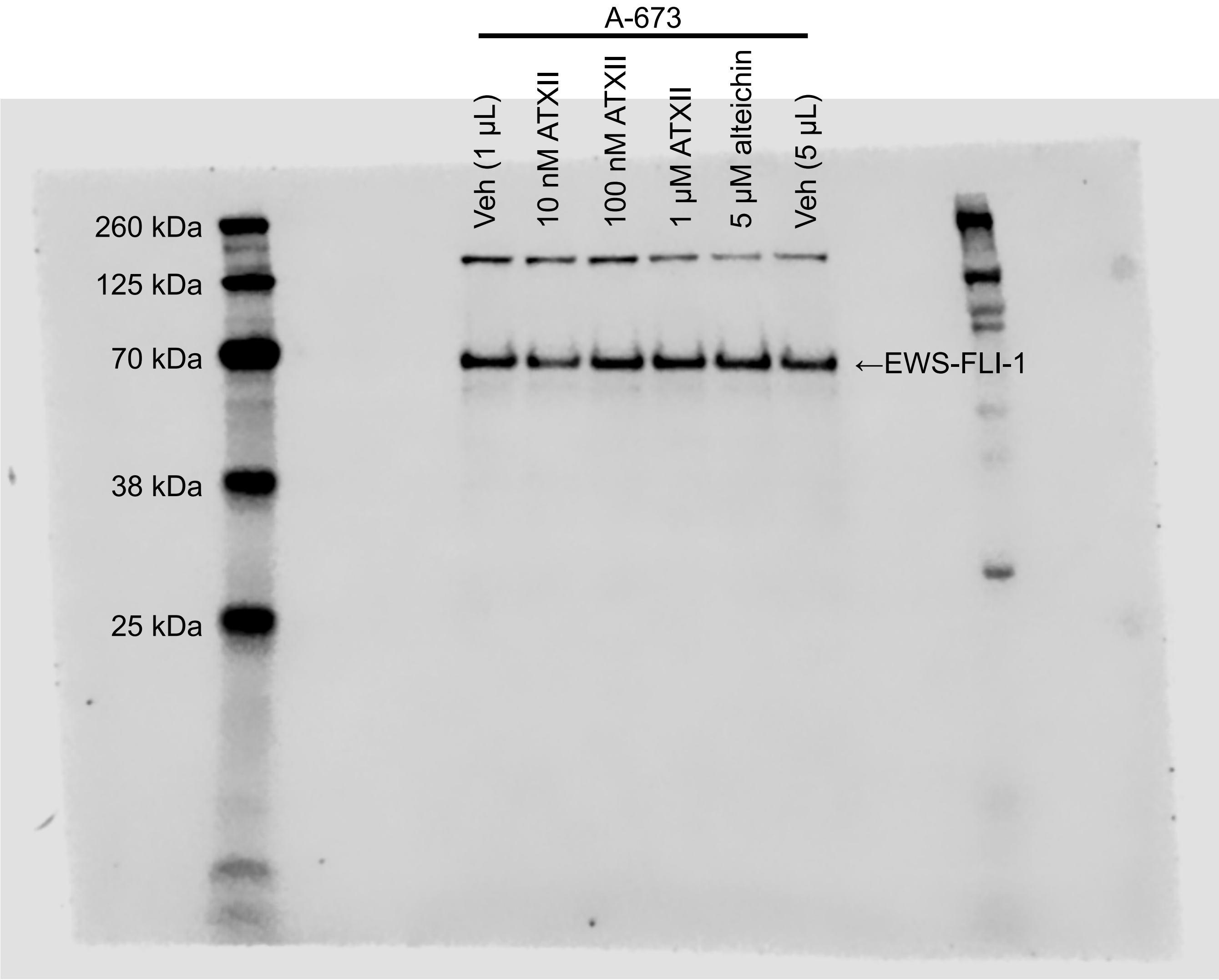

Figure 5A – P-S345-Chk1

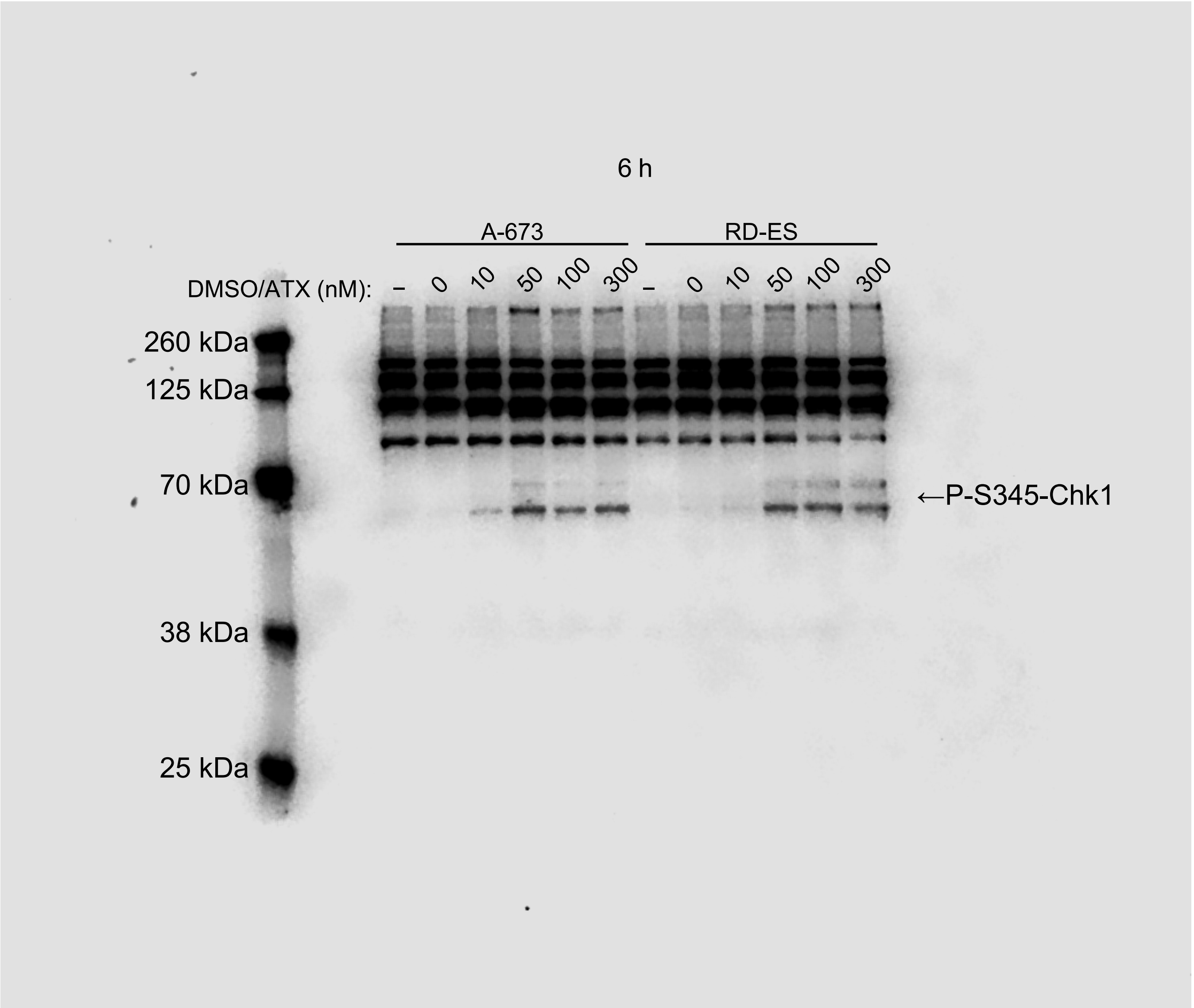

Figure 5A – Chk1

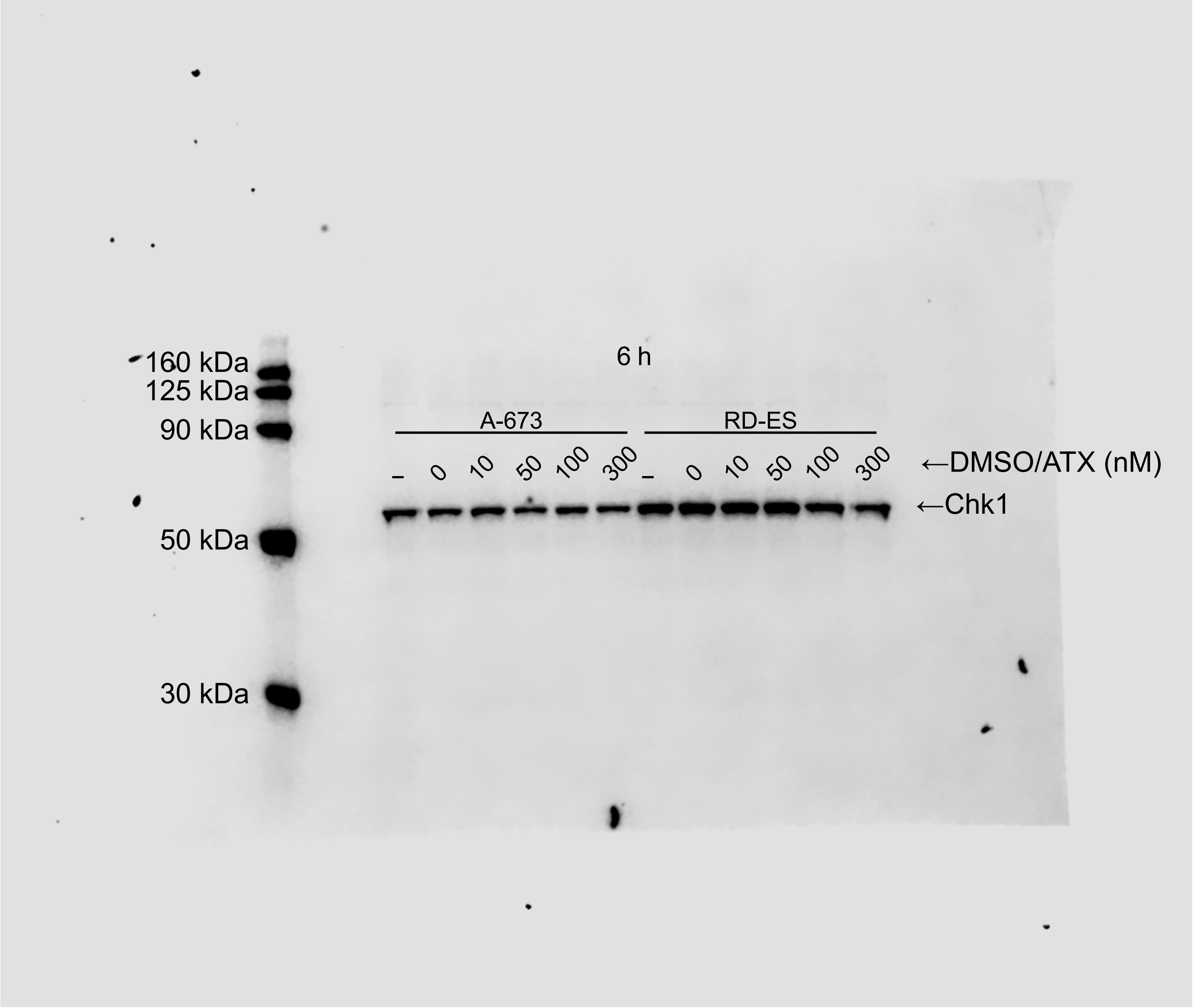

Figure 5A – P-T68-Chk2

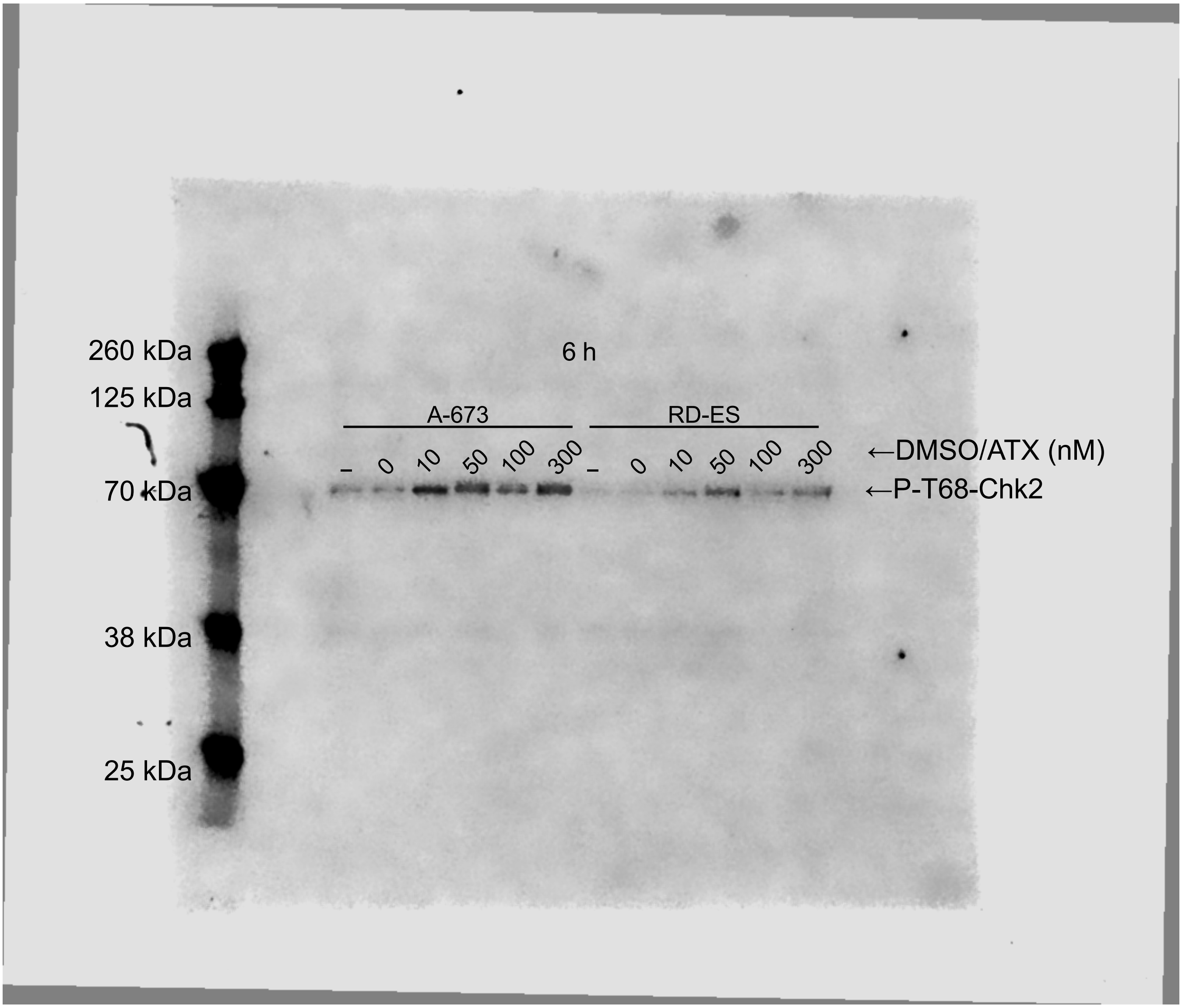

Figure 5A – Chk2

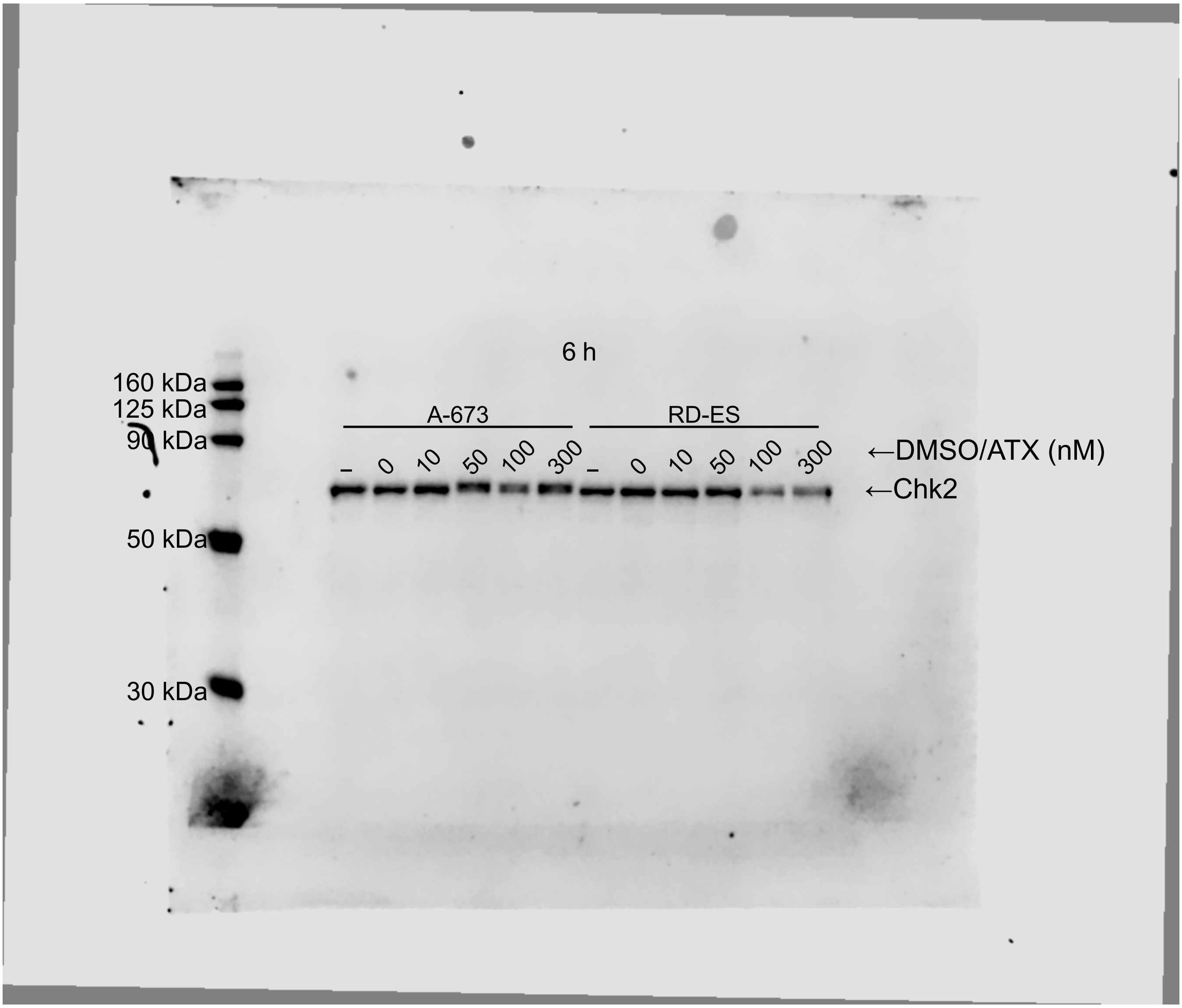

Figure 5A – P-S15-p53

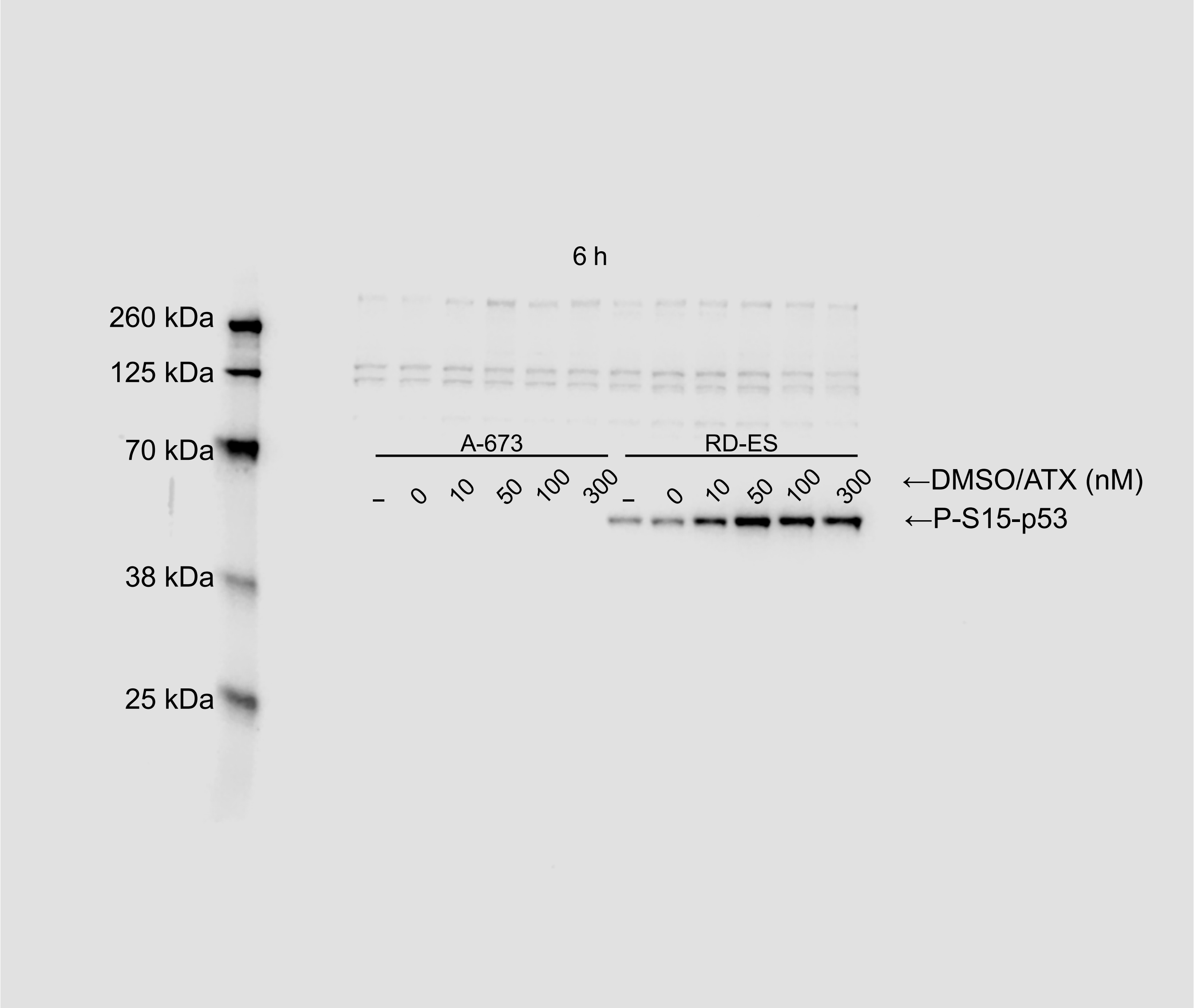

Figure 5A – p53

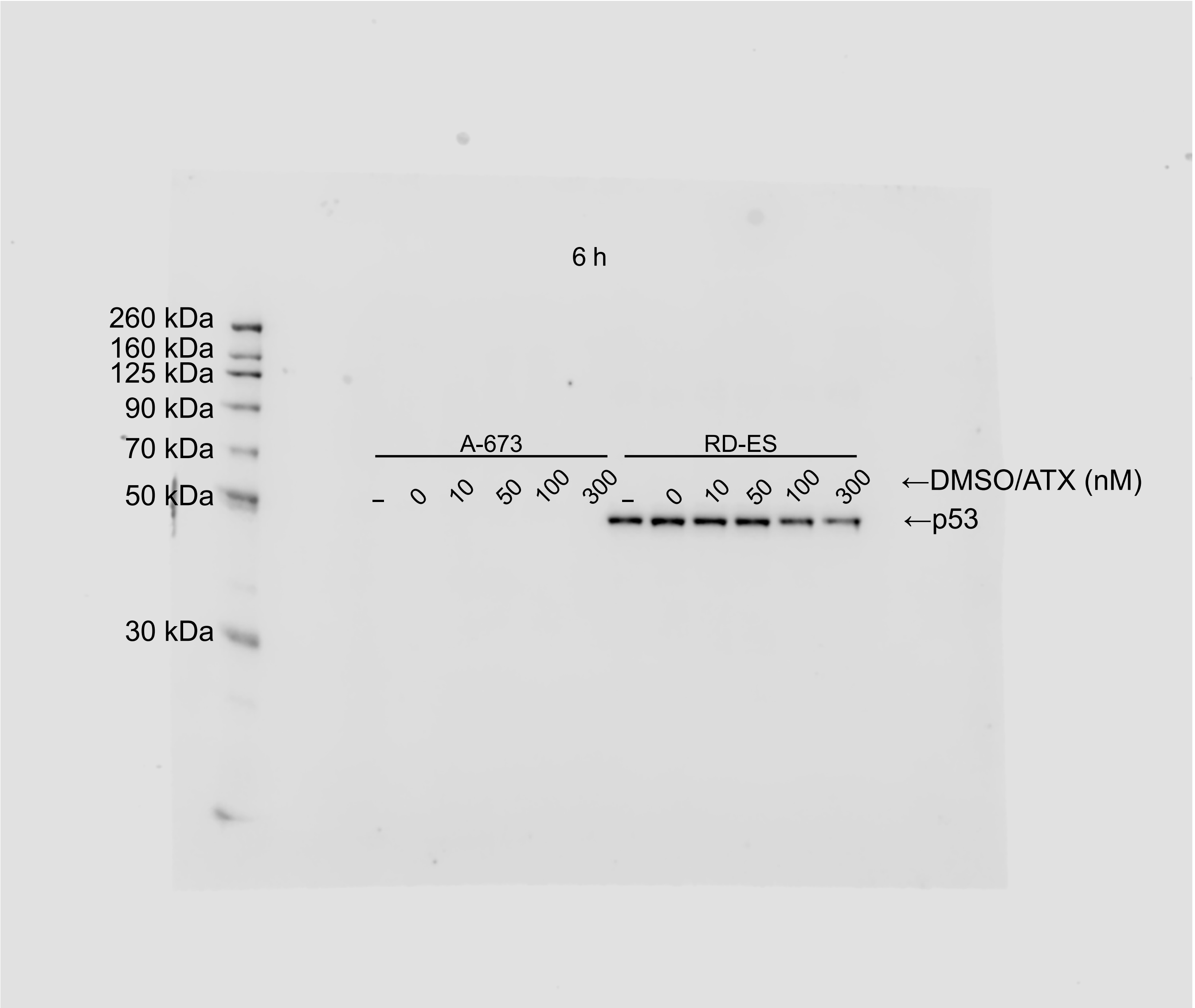

Supplement: Supplementary file 1 [file cancers-13-06176-s001.zip › WB Raw Images.pdf]
